# Recent Advances and Future Trends in the Detection of Contaminants by Molecularly Imprinted Polymers in Food Samples

**DOI:** 10.3389/fchem.2020.616326

**Published:** 2020-12-01

**Authors:** Mingkun Gao, Yuhang Gao, Ge Chen, Xiaodong Huang, Xiaomin Xu, Jun Lv, Jing Wang, Donghui Xu, Guangyang Liu

**Affiliations:** ^1^Key Laboratory of Vegetables Quality and Safety Control, Laboratory of Quality & Safety Risk Assessment for Vegetable Products, Ministry of Agriculture and Rural Affairs of China, Institute of Vegetables and Flowers, Chinese Academy of Agricultural Sciences, Beijing, China; ^2^Key Laboratory of Agro-Product Quality and Safety, Key Laboratory of Agro-Product Quality and Safety, Institute of Quality Standard and Testing Technology for Agro-Products, Chinese Academy of Agricultural Sciences, Ministry of Agriculture Beijing, Beijing, China

**Keywords:** molecularly imprinted polymers, nanomaterials, detection, food safety, contaminants

## Abstract

Drug residues, organic dyes, heavy metals, and other chemical pollutants not only cause environmental pollution, but also have a serious impact on food safety. Timely and systematic summary of the latest scientific advances is of great importance for the development of new detection technologies. In particular, molecularly imprinted polymers (MIPs) can mimic antibodies, enzymes and other biological molecules to recognize, enrich, and separate contaminants, with specific recognition, selective adsorption, high affinity, and strong resistance characteristics. Therefore, MIPs have been widely used in chemical analysis, sensing, and material adsorption. In this review, we first describe the basic principles and production processes of molecularly imprinted polymers. Secondly, an overview of recent applications of molecularly imprinted polymers in sample pre-treatment, sensors, chromatographic separation, and mimetic enzymes is highlighted. Finally, a brief assessment of current technical issues and future trends in molecularly imprinted polymers is also presented.

## Introduction

Food safety issues causing poisoning or death have become a major concern worldwide. Chemical pollutants, such as agricultural pesticides (Xiao et al., 2019), veterinary drug (Zhou et al., 2018), persistent organic pollutants (Ren et al., 2018), dyes (Im et al., 2017), and heavy metals (Rudd et al., 2016), usually have the characteristics of micro-toxicity, carcinogenesis, refractory degradation, and bioaccumulation (Liu X. et al., 2018; Rutkowska et al., 2018; Tarannum et al., 2020). Contaminants can enter the body through the food chain to cause skin, breathing, gastrointestinal tract, or systemic reactions that can lead to deadly anaphylactic shock that seriously threatens people's health (Ashley et al., 2017; Ghanbari and Moradi, 2017). At present, traditional detection techniques for ultra-low concentrations of chemical pollutants in complex samples include gas chromatography (GC), high performance liquid chromatography (HPLC) and other advanced technologies (Jafari et al., 2009). Although these methods have high sensitivity and good reproducibility, the chromatographic analysis technology still has the disadvantages of expensive laboratory sample pretreatment, complicated equipment, cumbersome sample purification and preparation steps, long processing time, and high requirements for personnel training, which limit its application (BelBruno, 2019). Therefore, the development of new sample pretreatment and rapid detection technologies, accurate quantification of chemical pollutants, effective identification of pollution levels, and food safety are of important research value and significance (Lu et al., 2015; Carvalho, 2017; Piletsky et al., 2020).

In recent years, researchers have proposed to combine molecularly imprinted polymers (MIPs) with conventional detection methods and improve them to obtain highly selective and sensitive detection strategies. Molecularly imprinted polymers are molecules that selectively bind to templated molecules in manufacturing processes through a “lock-and-key” mechanism, involving analytical chemistry, biology, and polymeric materials (Wulff, 2002; Han et al., 2016). In particular, it attracted much attention in the 1977 after Wulff et al. (1977) reported molecular imprinting technology, which uses a specific target molecule as a template to bind to the monomeric form of MIPs. After removal of the template molecule and cross-linking, MIPs have selective recognition sites that are completely complementary to the template molecule in terms of shape, size, and functional group (Huang et al., 2018). Briefly, the synthesis of MIPs mainly involves three steps: (1) Template molecules and functional monomers combine via covalent or non-covalent interactions to form complexes (Figueiredo et al., 2016). (2) The composite is immobilized by adding crosslinking and pore-forming agents (Kupai et al., 2017). (3) The template molecules are eluted, leaving behind polymer template structures matching the target molecules in shape and structure (Ansell and Mosbach, 1998). Therefore, MIPs are highly selective for those target molecules or structural analogs. The target molecules are recognized via hydrophobic interactions, hydrogen bonding, van der Waals forces, or electrostatic interactions. MIPs have the characteristics of structure-activity predictability, specific recognition, and wide applicability (Huang et al., 2011; Sharma et al., 2012; Song et al., 2014; Wei et al., 2015; Lulinski, 2017; Ren et al., 2018; Yuan et al., 2018b; Erturk Bergdahl et al., 2019).

**Figure 1:**
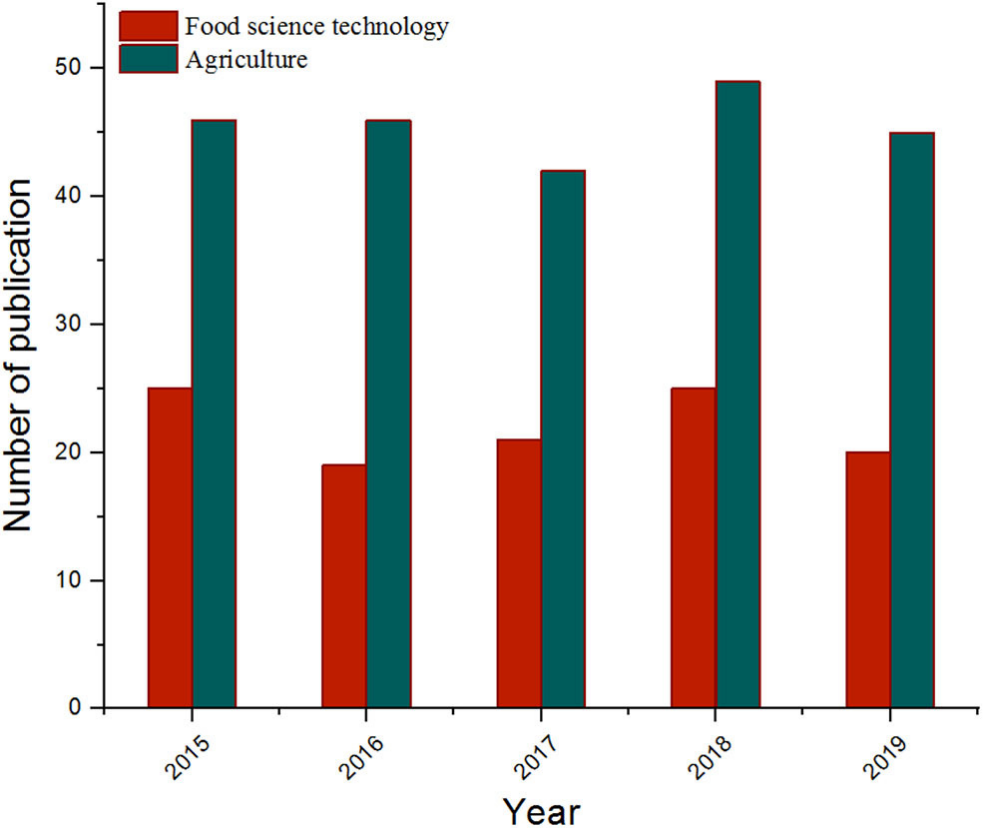
The growth trend of published articles on MIPs in food science technology and agriculture during 2015–2019 (data collected from Web of Science).

Figure 1 shows that the number of articles on MIPs published in the field of food science and technology and agriculture has been steadily increasing over the past 5 years, and it is very important to summarize the latest progress in this field in a timely and systematic manner to promote scientific progress. This review first describe the basic principles and production processes of molecularly imprinted polymers. Secondly, an overview of recent applications of molecularly imprinted polymers in sample pre-treatment, sensors, chromatographic separation, and mimetic enzymes is highlighted. Finally, a brief assessment of current technical issues and future trends in molecularly imprinted polymers is also presented. This research provides the necessary foundation for promoting the integration of MIPs and multidisciplinary technologies in the future, as well as for further development of MIPs with multifunctional applications.

## The Synthesis Process of MIPs

The synthesis of MIPs is the most common method of production. Briefly, functional monomers interact with target molecules in solution to form a network of complexes with covalent or non-covalent interactions. Based on the rearrangement process between the target molecule and the functional monomer in the polymer, it can be mainly classified into three types of interactions: covalent, non-covalent, and semi-covalent (Canfarotta et al., 2018; Huang et al., 2018; Sposito et al., 2018; Wang H. et al., 2018; Bagheri et al., 2019).

(1) In covalent imprinting, reversible covalent bonds between template molecules and functional monomers are usually used to form stable polymers. The main advantage of this technique is to obtain a very uniform distribution of binding sites in the polymer (Chen et al., 2017).

(2) Non-covalent imprinting is the most common type of interaction and relies mainly on the formation of weak binding interactions between the functional monomers and the template in the pre-polymerized mixture, such as hydrophobic or hydrogen bonding, dipole, and ionic interactions (Ashley et al., 2017).

(3) Semi-covalent imprinting techniques include both covalent and non-covalent imprinting processes, and although the molecular imprinting process is polymerized in the form of covalent bonds, interestingly the target molecule binds to the monomer with non-covalent interactions (Ansari, 2017).

**Figure 2:**
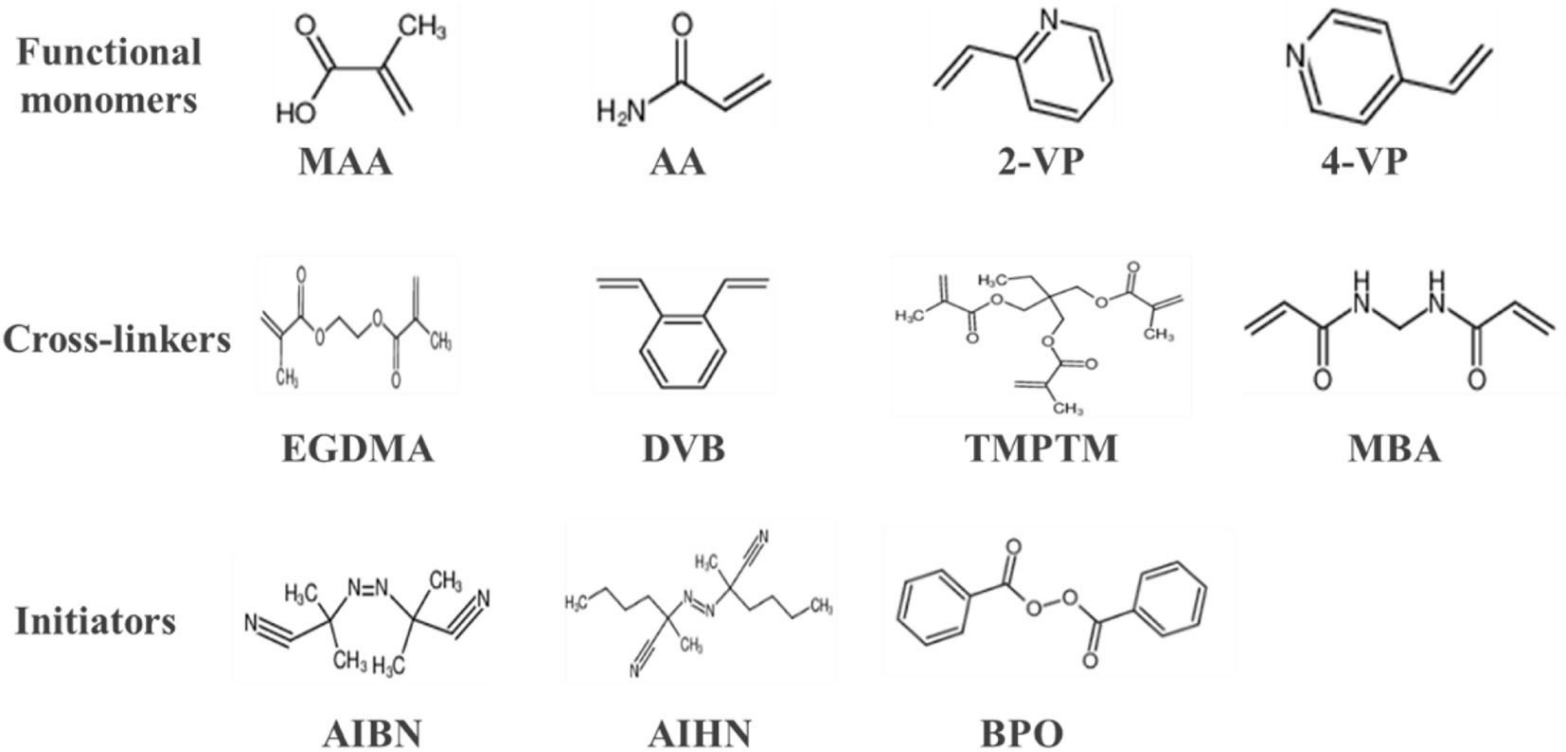
Some commonly used MIPs reaction reagents involving functional monomers, cross-linkers, and initiators. MAA, methacrylic acid; AA, acrylic acid; 2-VP, 2-vinyl pyridine; 4-VP, 4-vinyl pyridine; EGDMA, ethylene glycol dimethacrylate; DVB, divinylbenzene; TMPTM, trimethylolpropane-tri methacrylate; MBA, methylene acrylamide; AIBN, azobisisobutyronitrile; AIHN, azodiisopentanyl; BPO, benzoyl peroxide.

**Figure 3:**
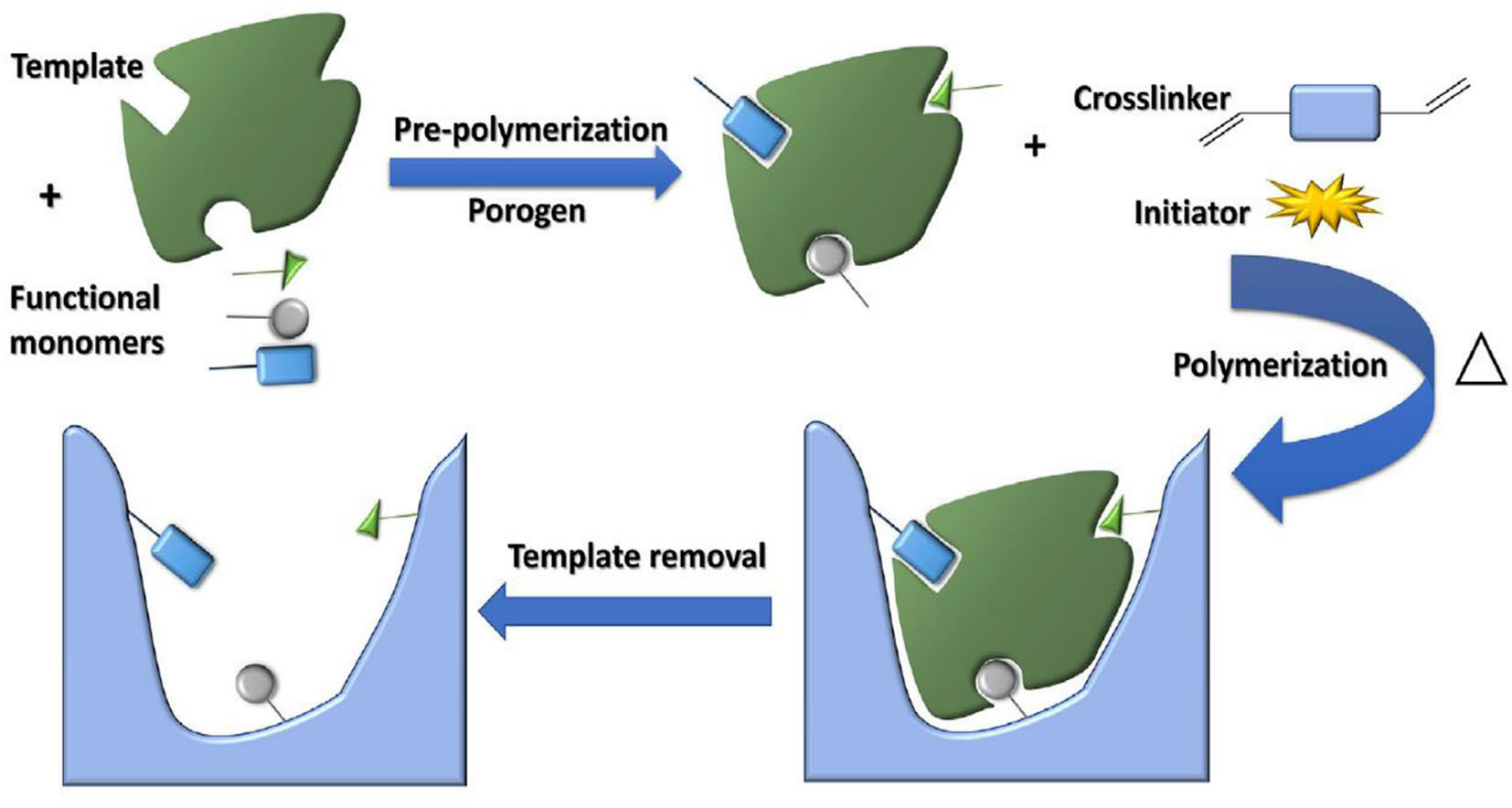
The schematic diagram of MIPs synthesis (Azizi and Bottaro, 2020). Reprinted with permission from Elsevier. Copyright (2020) Elsevier.

Functional monomers, crosslinkers and initiators are the three basic elements in the synthesis of MIPs, and some commonly used functional monomers, initiators, and crosslinkers are shown in Figure 2. Functional monomers play an important role in the synthesis of all molecularly imprinted polymers by forming specific complexes with templates through covalent or non-covalent interactions. In fact, the functional hydrogen bonds or active substituents provided by the functional monomers not only affect the affinity of the template to the functional monomer molecules, but also determine the mechanical stability and porosity of the polymer (Figueiredo et al., 2016; Singh et al., 2020). The preparation of molecularly imprinted polymers involves the formation of a complex when the functional monomer reacts sufficiently with the template molecule, followed by the addition of a cross-linking agent to immobilize the functional group of the functional monomer onto the imprinted molecule. Finally, a highly cross-linked rigid polymer is formed even after removal of the template (Chen et al., 2016). Figure 3 shows a typical process for the synthesis and recognition of MIPs. Typically, too low an amount of crosslinker decreases the structural stability of the polymer, leading to the shedding of functional monomers. In addition, an excessive amount of cross-linking agent reduces the number of sites recognized by MIPs. Currently, the vast majority of commercial cross-linkers are compatible with molecular imprinting, and a few have the ability to complex with templates, such as ethylene glycol dimethacrylate (EGDMA). Currently, initiation methods can be categorized as thermal, photo,- and electrical initiation. The involvement of the initiator is required to ensure that the molecularly imprinted polymer proceeds as usual and to shorten the reaction cycle. This may even affect the material properties.

## Method of Synthesis of MIPs

**Figure 4:**
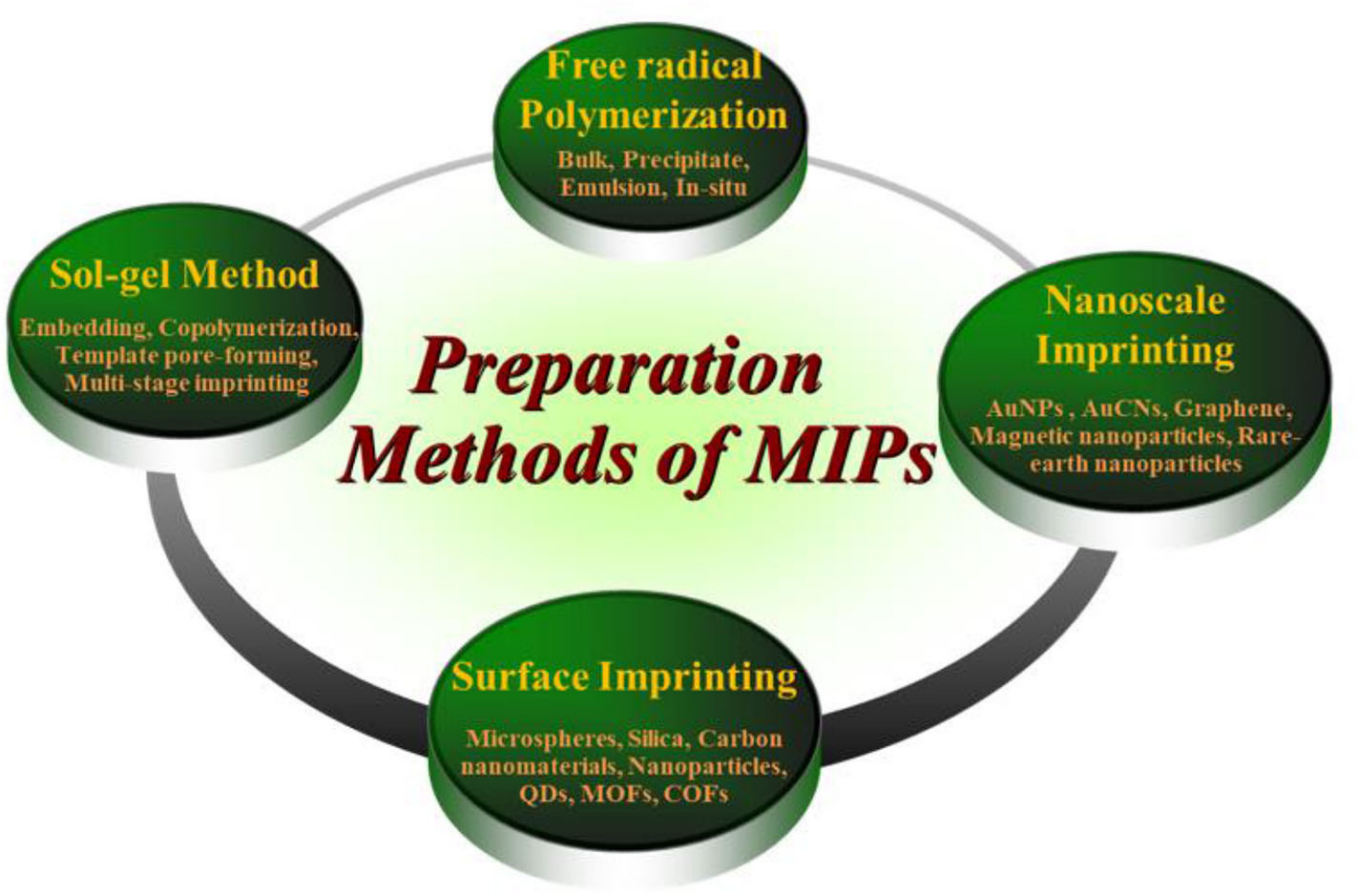
Preparation methods of MIPs based on different polymerizations.

The increasing refinement of functionalized nanoparticles provides infinite possibilities for the preparation of MIPs. There are many approaches leading to the preparation of MIPs with different host-polymer properties, which can be broadly divided into three approaches: (1) synthesis with the aid of functional monomers with the involvement of templates in the reaction process (2) interconversion using polymer precipitates by introducing incompatible solvents or by evaporation of solvents from solution (3) obtaining polymers by means of soft lithography or surface stamping. The methods of preparation of MIPs based on different polymerization methods are shown in Figure 4. The methods evident in the figure are not exhaustive, but only present possible routes for MIP preparation. Other examples are also discussed in the text. Currently, how to continuously improve the methods and optimize the conditions by incorporating nanoparticles has become the focus of research for the preparation of new MIPs.

### Sol-Gel Method

The sol-gel method is one of the most common methods for the preparation of molecularly imprinted polymers due to its intrinsic conversion efficiency and material homogeneity at the molecular level, high solvent, and thermal stability, and one-step preparation process (Beqqali et al., 2017; Guoning et al., 2020). The most common sol-gel method uses methyl orthosilicate (TMOS) or ethyl orthosilicate (TEOS) as a precursor to introduce the imprinted template into the inorganic network structure, which is homogeneously mixed in the solution state and subjected to hydrolysis and condensation chemistry to form highly stable chemically-reacted crosslinked polymers (Dai et al., 2014; Figueiredo et al., 2016; Barsbay and Güven, 2018). The three most notable features of the sol-gel method include (1) Mild operating conditions and simple preparation methods (Liu and Wulff, 2008). (2) The porosity and surface area of the polymer powder/film can be controlled (Wei et al., 2015). (3) The selectivity of the material and the functional units in the proprietary network structure can be significantly improved by the introduction of specific chemical substances (Ma et al., 2015). In order to obtain functionally diverse molecularly imprinted polymer materials, in this section we focus on the modification of polymer materials by introducing different chemical substances to thereby achieve detection and adsorption effects.

**Figure 5:**
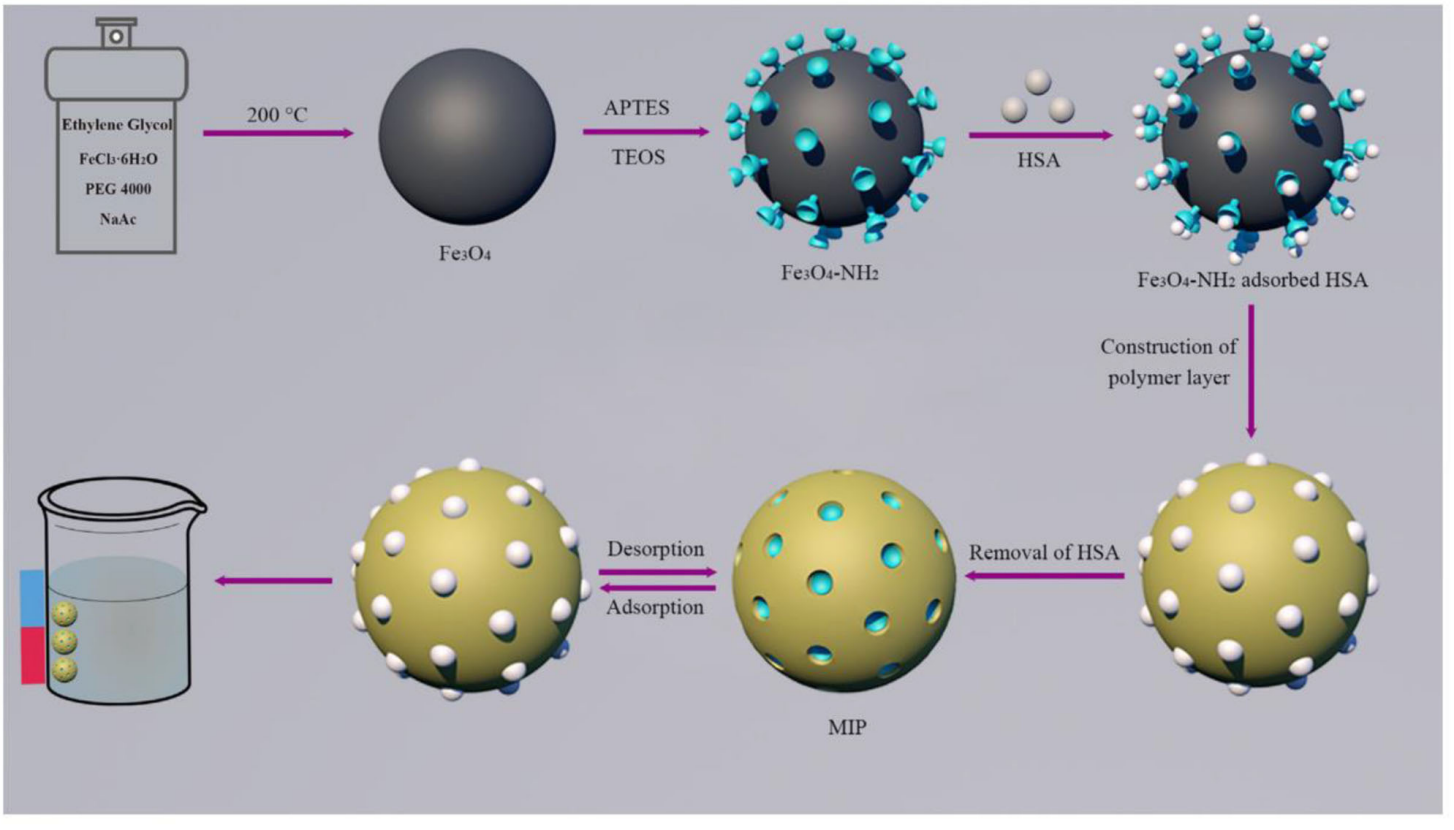
The principle and procedure for the preparation of MIPs (Guoning et al., 2020). Reprinted with permission from Elsevier. Copyright (2020) Elsevier.

Guoning et al. (2020) designed a surfactant-mediated sol-gel system to construct imprinted polymer layers for the preparation of protein MIPs (Figure 5), in which 3-(methacryloxy) propyltrimethoxysilane (MPS) was used as a cross-linker monomer and solvent-containing surfactant was used as a mild hydrolysis system. In this reaction system, a homogeneous solution was obtained, avoiding the disruption of protein structure in the organic solvent environment. The Fe_3_O_4_ nanospheres obtained by grafting the imprinted polymer layer onto the surface of the carrier nanoparticles using a surfactant-mediated sol-gel method can undergo rapid magnetic separation and immobilization under an applied magnetic field. Finally, because the large number of non-specific binding sites in MIPs and the abundance of functional groups on the protein surface lead to unavoidable cross-reactions, a blocking strategy was introduced to reduce non-specific cross-reactions and increase selectivity, which reduces the feasibility of MIPs as an alternative to immunoassay antibodies. Using the same approach without using a template, Li et al. (2013) synthesized novel titanium dioxide based MIPs by sol-gel method using sunset yellow as a template. MIPs were used as solid phase extraction materials for the separation and enrichment of sulfonic acid dyes in beverages. The results showed that the MIPs had better selectivity, higher recovery, and adsorption capacity for sulfonic acid dyes compared to non-imprinted polymers (NIPs). In addition, in the case of using functional monomers, Wang et al. (2013) prepared water-soluble MIPs suitable for solid-phase microextraction (SPME) using sol-gel technique with diazinon as template and polyethylene glycol as functional monomer. The limits of detection of organophosphorus pesticides (OPPs) measured ranged from 0.017 to 0.77 μg/kg. The method was applied to cucumber, green pepper, cabbage, eggplant, and The organophosphorus pesticides (OPPs) in lettuce samples were determined in the range of 81.2–113.5% recoveries for each vegetable using the standard addition method.

### Free Radical Polymerization

Free radical polymerization is initiated by monomeric free radicals containing unsaturated double bonds. Ontopolymerization is one of the most common and widespread methods of preparing MIPs by conventional free radical polymerization, which has the advantages of rapid and simple preparation without the need for complex and expensive instrumentation and purity. However, the whole polymer obtained by ontology polymerization must be crushed, ground, and sieved to the appropriate size, which significantly reduces production efficiency. In addition, grinding operations can result in irregular particle shapes and sizes, which can disrupt some of the high affinity binding sites. Ontopolymerization produces polymers with inhomogeneous binding site distributions, which greatly limits the use of MIPs as chromatography adsorbents. To overcome these drawbacks of ontology polymerization, various attractive polymerization strategies such as suspension polymerization (Zhou et al., 2018), emulsion polymerization (Abdollahi et al., 2018), seed polymerization (Kong et al., 2013; Ma et al., 2015), and precipitation polymerization (Rodriguez et al., 2019) have been proposed. A more uniform distribution of binding sites should be obtained since the post-treatment steps required in ontology polymerization are not required when alternative polymerization strategies are used. In the aforementioned polymerization methods, MIPs are typically prepared by conventional suspension polymerization in the presence of a stabilizer or surfactant and water is used as a continuous phase to suspend the droplets of the pre-polymerization mixture. At the same time, radical polymerization suffers from the disadvantage that the polymer binder is prone to dislodge from the template; therefore, atom transfer radical polymerization (ATRP) and reversible addition-breakage chain transfer (RAFT) polymerization are considered to be the most promising methods for the preparation of MIPs compared to conventional radical polymerization. In 2018, Söylemez's group (Söylemez et al., 2018) prepared atrazine by methacrylic acid (MAA) using RAFT polymerization and using dithiobenzoic acid (CDB) and ethylene glycol dimethacrylate (EGDMA) as the RAFT agent and crosslinking agent, respectively. It was demonstrated that The MIPs synthesized by the RAFT method presented an increase of over 100% in binding capacity at low atrazine feed concentrations compared to those prepared by conventional free-radical polymerization method where no RAFT agent was employed. Also besides, Barsbay and Güven (2018) reported the synthesis of atrazine MIPs polymers RAFT precipitation polymerization methods. The imprinting transfer efficiency of atrazine MIPs was significantly improved compared with the MIPs prepared by conventional precipitation polymerization RAFT-MIPs with spherical spatial structure and a large number of micropores on the surface.

Studies of selectivity and reusability are important for molecularly imprinted polymers, for example: Wang Z. et al. (2018) constructed a molecularly imprinted polymer@silver nanoparticle surface-enhanced Raman scattering (SERS) sensor by *in situ* preparation of silver nanoparticles in a bisphenol a molecularly imprinted polymer matrix. The *in situ* formed silver nanoparticles were uniformly distributed and tightly stacked in the matrix, which facilitated hotspot formation and analyte-silver nanoparticle affinity. The molecularly imprinted polymer@silver nanoparticle sensor shows good bisphenol a selectivity for structurally related molecules such as bisphenol AF (BPAF) and diethylstilbestrol (DES).This MIPs@AgNPs sensor has excellent sensitivity and a detection limit of at least 5 × 10^8^ mol/L for BPA. This SERS sensor is easily regenerated by solvent washout and Sodium borohydride reduction was originally realized. This molecularly imprinted polymer@silver nanoparticle SERS sensor has the advantages of simple fabrication, selective identification, high sensitivity, and reusability, and has promising applications. Recently. Arias et al. (2020) synthesized a selective molecularly imprinted solid-phase extraction sorbent and applied it to the extraction of chlorpyrifos, diazinon, and their corresponding oxo forms from aqueous samples, followed by HPLC-UV analysis. Several parameters affecting the extraction of the imprinted polymer, such as the composition and volume of the washing solvent, the elution solvent, and the sample volume, were studied, and under optimal conditions, the method had a detection limit of 0.07–0.12 μg/L and the material had good reusability (more than 50 times). The average recoveries were 796–1,043%. This study shows that molecularly imprinted polymers prepared with diazinon as template molecule have the best recognition ability and significant affinity for these compounds. Therefore, it is a promising alternative method for monitoring chlorpyrifos, diazinphos, and their oxo forms in water samples.

### Surface Imprinting

MIPs prepared by traditional polymerization methods often have the shortcomings of deeply embedded binding sites, incomplete template elution, facile template exudation, and slow mass transfer rates (Turiel and Martin-Esteban, 2010). Recently, MIPs have been modified on the surface or the outer layer of a specific carrier, so that most of the specific binding sites are externally distributed. This is conducive for the removal and recombination of template molecules, it reduces the “embedding” phenomenon and migration resistance of template molecules, and it improves both the amount of adsorption and the MIP_S_ mass transfer rate (Carter and Rimmer, 2004; Li et al., 2006). Commonly used carriers are polystyrene microspheres (Sonawane and Asha, 2017), silica (Wang et al., 2017), carbon nanomaterials (Dai et al., 2014), magnetic nanoparticles (Ning et al., 2017), quantum dots (Yu et al., 2017), and metal-organic frameworks (Zhang et al., 2016). The surfaces of imprinted materials are easy to control, and the molecularly imprinted sites are readily exposed to targets. Template molecules can be eluted completely and have low migration resistance when they are adsorbed selectively, which overcomes the embedding phenomenon. The specific surface area is large, the density of imprinting is high, and the adsorption capacity and efficiency are high.

**Figure 6:**
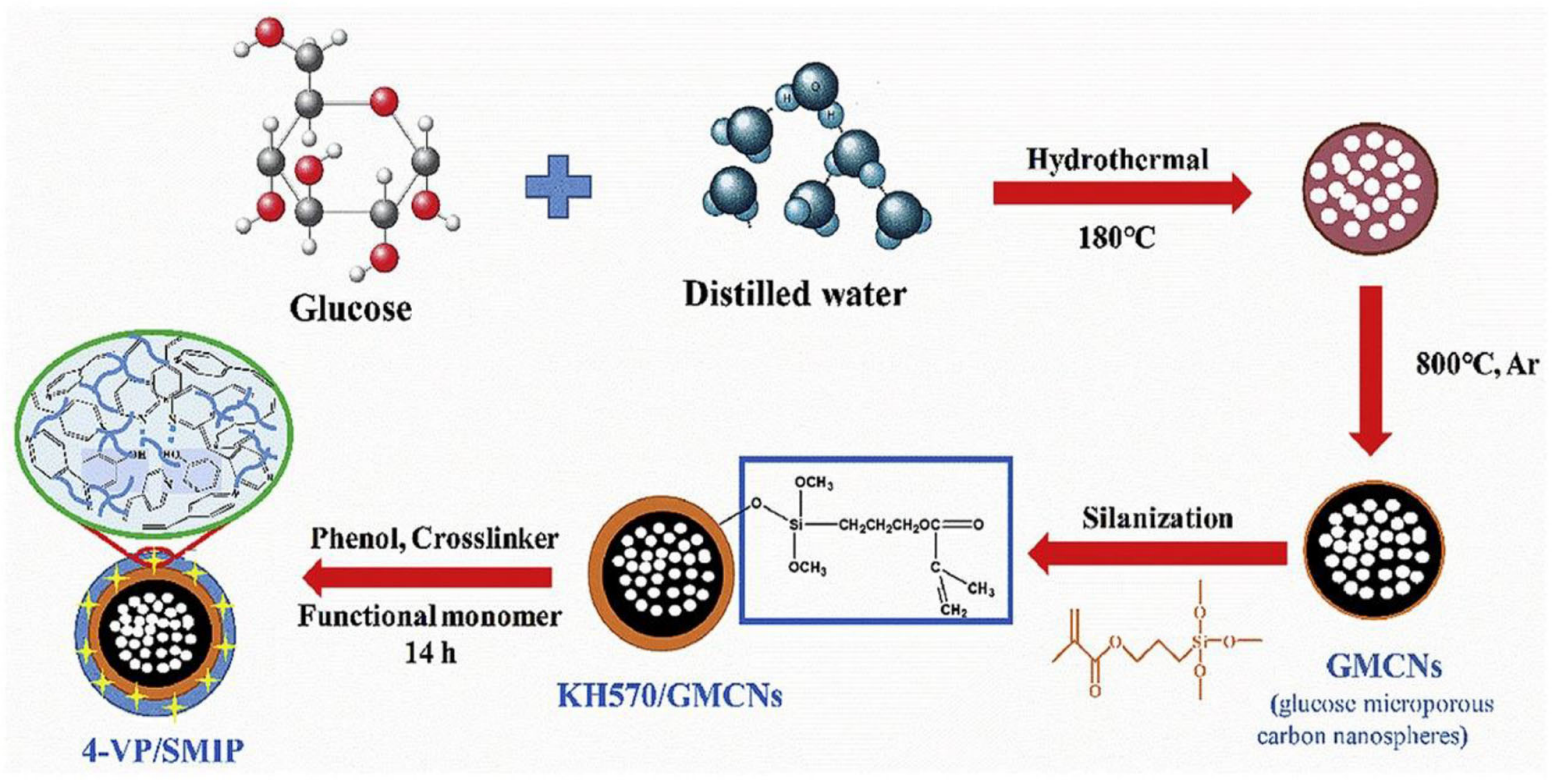
Schematic diagram of the synthesis of surface molecularly imprinted polymers based on glucose-derived microporous carbon nanoparticles (Qu et al., 2020). Reprinted with permission from Elsevier. Copyright (2020) Elsevier.

Recently, a highly selective surface molecularly imprinted polymer (SMIP) was prepared on glucose-derived microporous carbon nanospheres (GMCNs) for the removal of phenol from wastewater by surface molecular imprinting technique was reported. Qu et al. (2020) used GMCNs with abundant pore structure and surface oxygen-containing functional groups as the carrier material, and the active layer was constructed by grafting the silane coupling agent 3-(methacryloyloxy) propyltrimethoxysilane, and the schematic of the preparation procedures was presented in Figure 6. The results showed that the excellent adsorption capacity and selectivity of 4-VP/SMIP provided a feasible method for the effective separation of phenol from wastewater. Similarly, Liu et al. (2015) prepared magnetic MIPs (MMIPs) with good specificity and high adsorption capacity via surface molecular imprinting with magnetic C_3_N_4_ nanoparticles as carriers and atrazine as a template. The surface of the polymer was rough, the average particle size was 2 μm, the magnetic properties were remarkable, and it exhibited highly selective recognition of atrazine pesticides. Qian et al. (2016) prepared molecularly imprinted thin layers on MOFs via surface imprinting and then modified the surface with a layer-by-layer self-assembly technique for the electrochemical detection of methomyl pesticide residues in pears. The linear range was 0.1–0.9 mg/L, and the detection limit was as low as 0.0689 mg/L.

### Nanoscale Imprinting

Nanoscale imprinting is based on surface imprinting. A nanoscale molecularly imprinted polymer (Jiang et al., 2017) can be prepared by introducing nanoscale materials during the synthesis process of imprinted materials, or by directly using the materials for imprinting. Because nanoscale materials can greatly increase the specific surface area and physicochemical properties, the detection sensitivity of MIPs sensors will be improved, the linear range will be wider, and the detection limit will be lower (Canfarotta et al., 2018). Fluorescence emission or electrochemical signals are possible by incorporating fluorescent or electrochemical nanomaterials in the MIP_S_ synthesis. Commonly used nanomaterials include gold nanoparticles (Figueiredo et al., 2016), quantum dots (Ensafi et al., 2017), carbon nanotubes (Akhoundian et al., 2019), graphene (Duan et al., 2016), magnetic nanoparticles (Piletska et al., 2015), and rare-earth nanoparticles (Liu et al., 2017). Many improve the sensitivity via high specific surface areas, adjustable surface properties, and high electrical conductivity.

**Figure 7:**
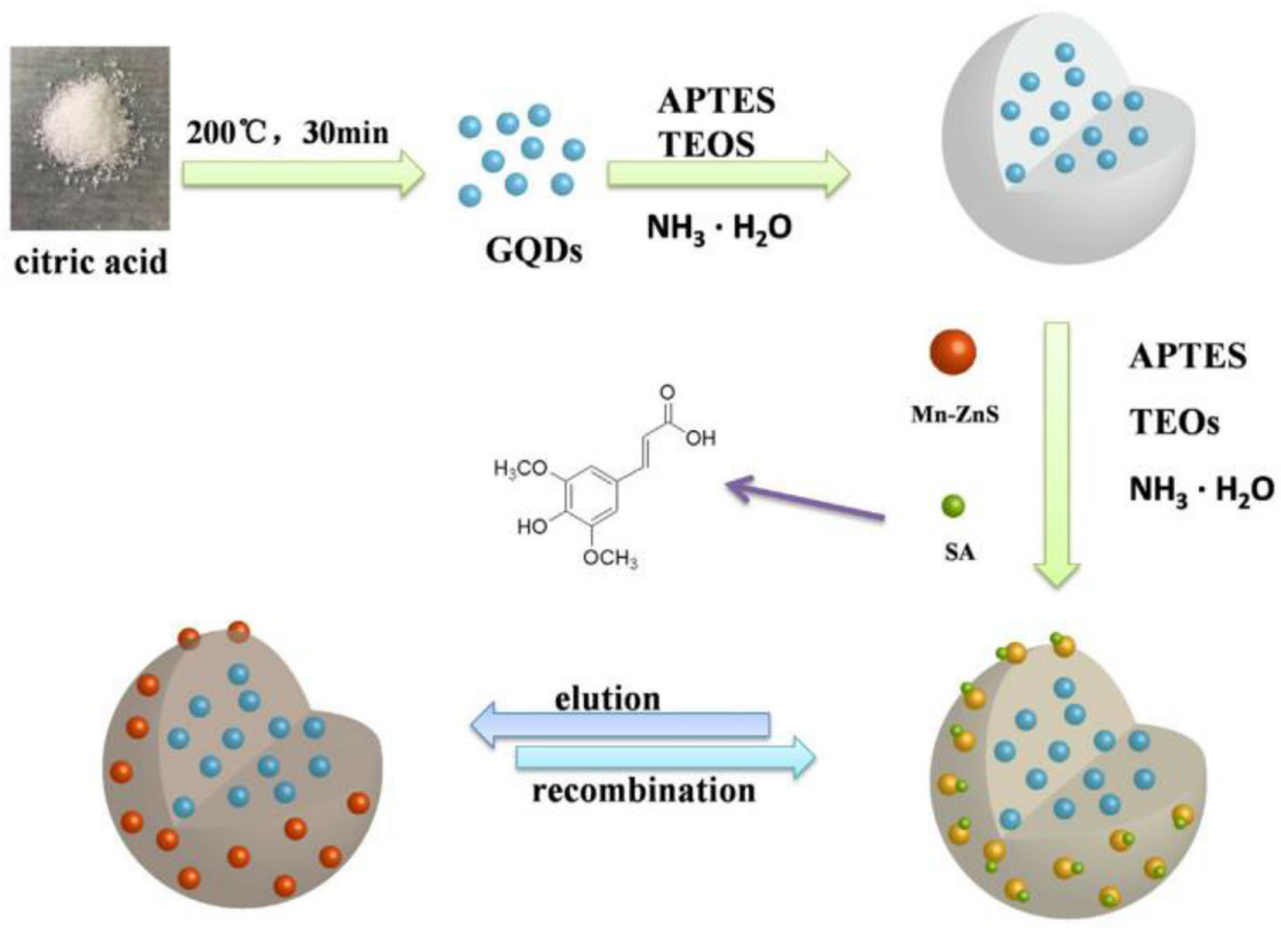
Schematic illustration for the preparation of the ratiometric fluorescence sensors based on MIPs@Mn-ZnS/GQDs@SiO_2_ and for the SA detection and recognition (Zhu et al., 2020). Reprinted with permission from Elsevier. Copyright (2020) Elsevier.

Zhao et al. (2017) synthesized novel MIPs from silica-modified multi-wall carbon nanotubes (MWNTs@SiO_2_) by combining surface MIPs with the sol-gel method. Using phenol as a template, 3-aminopropyltriethoxysilane as a functional monomer, and tetraethoxysilane as a crosslinking agent, the MIPs were grafted onto the surface of an MWNT. The maximum imprinting factor was 3.484. For the detection of 1, 4-dihydroxyanthraquinone, Nezhadali et al. (2016) prepared molecularly imprinted thin films on a carbon nanotube-modified carbon electrode via electropolymerization with pyrrole as functional monomer. Similarly, Zhu et al. (2020) established a sensitive, selective, and visual detection method for erucic acid (SA) based on Mn-ZnS-QDs and silica-coated graphene quantum dot molecularly imprinted polymers (MIPs) coated with double quantum dots (QDs) as a functional ratio fluorescence sensor (Figure 7). The polymers were synthesized by a simple one-pot sol-gel reaction with two fluorescence emission peaks at 580 nm, yellow fluorescence at the Mn-ZnS quantum dot, and blue fluorescence at 445 nm. SA selectively enhances the fluorescence of the quantum dots, but bursts the fluorescence of the Mn-ZnS quantum dots with MIPs@Mn-ZnS/GQDs@SiO_2_. The ratio of reduction was linearly related to SA concentration in the range of 9–81 nm, with a detection limit of 0.8388 nm (S/N = 3). The constructed fluorescent probe can also be visually detected based on color change.

## Polymerization Techniques

### Dummy MIPs

Molecularly imprinted polymers were used in sample pretreatment in early research, but with the development of research, many obstacles were created. For example: (1) The target analyte is consumed too much directly as a molecular template. (2) Functional templates and functional monomers do not have good stability during sample preparation. Nevertheless, in 1997, Andersson's group (Andersson et al., 1997) first used a tight structural analog to replace the real target molecule as a template. subsequently, In 2000, Haupt and Mosbach (2000) proposed the virtual molecule based on the concept of imprinted polymers, today, virtual molecularly imprinted polymers play an important role in the synthesis strategy of imprinted polymers.

**Figure 8:**
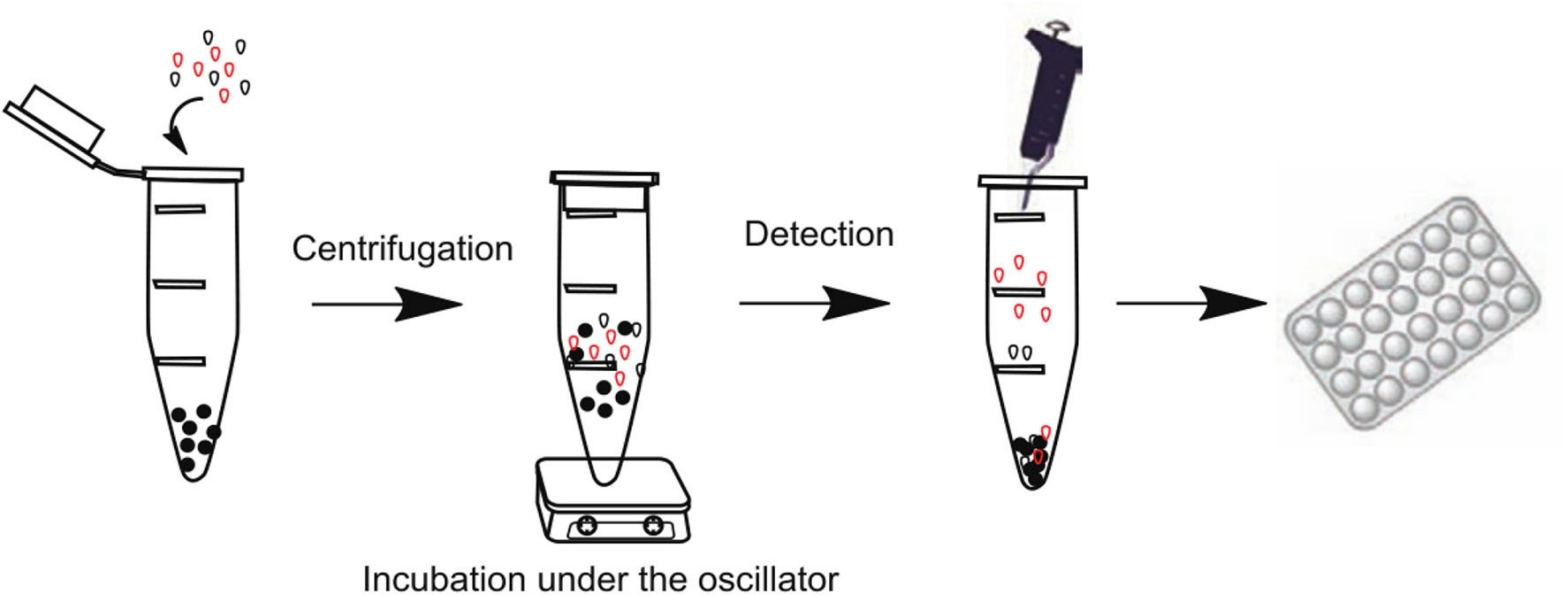
Schematic diagram of DMIP-Trimel Fluorescence Competitive Analysis (Du et al., 2016). Reprinted with permission from Elsevier. Copyright (2016) Elsevier.

Bagheri et al. (2019) prepared a novel dummy molecularly imprinted polymers (DMIP) with propanamide as a dummy template molecule based on a green synthesis strategy of less consumption of hazardous/organic reagents for magnetic solid-phase extraction (MSPE) of acrylamide in biscuit samples. The method recoveries at five spiked concentrations were found within 86.0–98.3% with relative standard deviations (RSD) of 1.2–4.1%. There are many more analytical tests for acrylamide, for instance: Arabi's group (Arabi et al., 2016a) applied a novel technique for the synthesis of dummy molecularly imprinted silica nanoparticles (DMISNPs), the material was used as a dispersant for the analysis of biscuit and bread samples using matrix solid-phase dispersion (MSPD). It was proven that the proposed dispersant leads to a high affinity toward acrylamide even in complicated matrices. Similarly, Arabi et al. (2016b) constructed a novel and facile preparation of magnetic virtual molecularly imprinted nanoparticles (MDMINP) for pre-enrichment of acrylamide in potato chips by Fe_3_O_4_ magnetic nanoparticles (MNPs) and APTMS. The particle has excellent magnetic properties and high selectivity to target molecules. Under optimal conditions, the recovery ranged from 94.0 to 98.0% with the detection limit of 0.35 μg/kg. Du et al. (2016) established a fluorescence competitive analysis method for melamine using pseudomolecularly imprinted polymers (DMIPs) as artificial antibodies, and the schematic of the preparation procedures was presented in Figure 8. The DMIPs used were synthesized under hot water bath conditions using 2,4-diamino-6-methyl-1,3,5-triazine (DAMT) as a pseudotemplate, methacrylic acid (MAA) as a functional monomer, ethylene glycol dimethacrylate (EGDMA) as a crosslinker, and azobisisobutyronitrile (AIBN) as an initiator, and the synthesized DMIPs had specific recognition sites for melamine. The best detection system showed a linear response in the range of 0.05–40 mg/L with a limit of detection (LOD) of 1.23 μg/L. The fluorescence competitive assay for MIPs as antibody mimics was validated to be less sensitive and specific than ELISA, but it achieved satisfactory accuracy, and MIPs also have the advantages of low cost, ease of synthesis, and high stability to harsh chemical and physical conditions.

### Magnetic Molecularly Imprinted Polymer

Although MIPs have been excellent, some drawbacks are evident, such as the lack of electrocatalytic and conductive properties of MIPs, in order to increase its conductivity and repeatability, and thus introduced magnetic molecularly imprinted polymer (MMIP) to immobilize nanomaterials as an effective way to update MIPs, the combination of magnetic nanoparticles and molecularly imprinted polymer prepared magnetic molecules. The imprinted polymer not only specifically identifies the target, but can also be rapidly separated from the substrate by the action of an applied magnetic field. MMIPs offer many superior properties compared to conventional MIPs, including simplicity of manipulation, rapid binding to the target analyte, magnetic susceptibility, and shorter pretreatment times. Recently, a number of methods have been improved for the detection of different compounds using magnetic electrodes.

**Figure 9:**
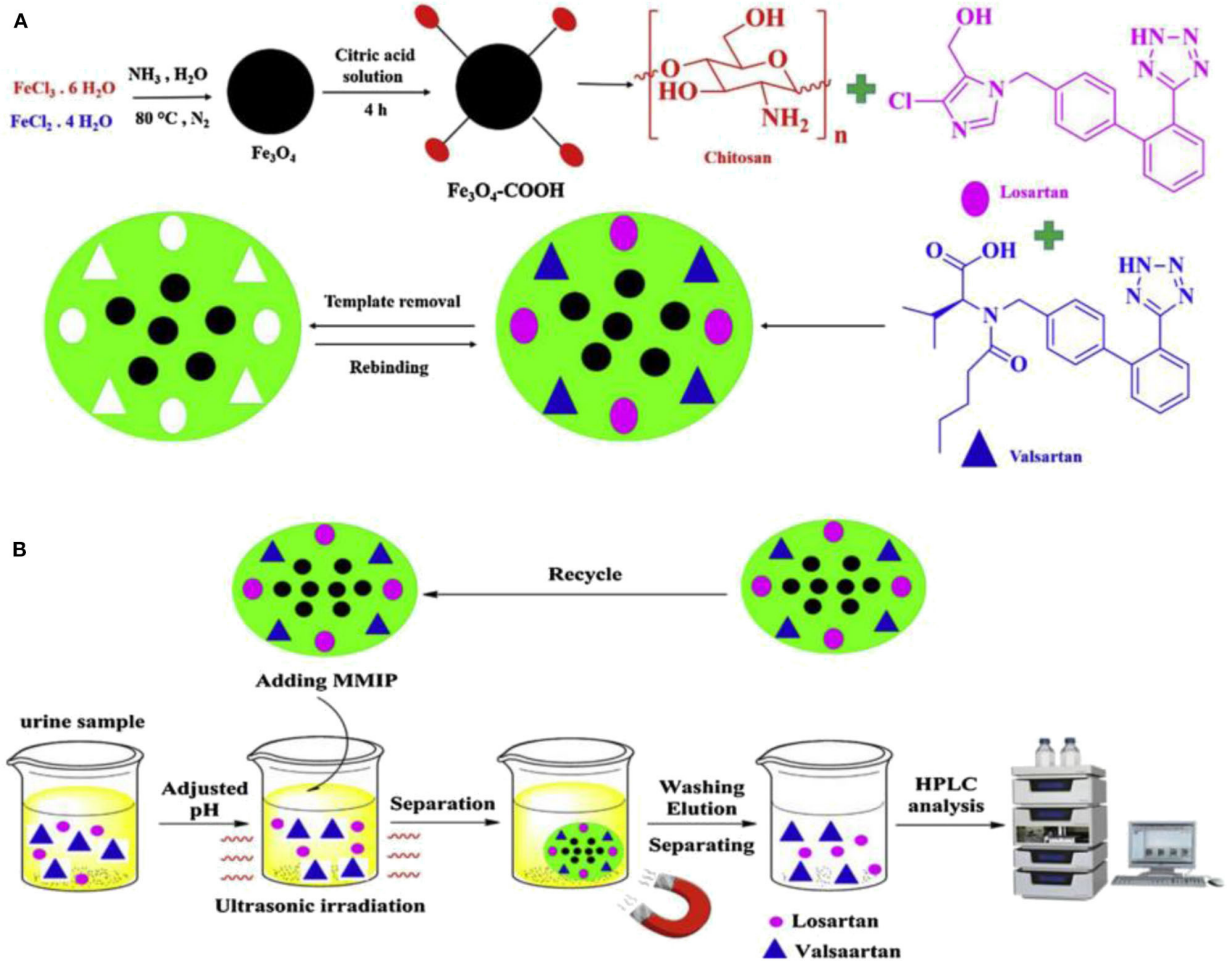
The basic preparation procedure of MMIP **(A)** and the MMIP based MDSPE procedure for valsartan and losartan extraction **(B)** (Bagheri and Ghaedi, 2020). Reprinted with permission from Elsevier. Copyright (2020) Elsevier.

Bagheri and Ghaedi (2020) prepared dual-template chitosan-based magnetic water-soluble molecularly imprinted biopolymers using a green synthesis method without the use of organic and toxic reagents for the simultaneous determination of urinary valsartan (VAL) and chlorosartan (LOS) adsorbents by high performance liquid chromatography-ultraviolet spectrophotometry (Figure 9). Chitosan is considered as a multifunctional monomer due to its unique properties such as non-toxic, inexpensive, readily available, biocompatible, biodegradable, and easy polymerization under mild conditions. The adsorbent has the advantages of green synthesis, high magnetic strength, biocompatibility, high selectivity, fast equilibrium adsorption, and high adsorption capacity. Similarly, Zeng et al. (2012) proposed a method for the preparation of magnetic molecularly imprinted polymers (MMIPs), which can be used for the separation and purification of rutin. The performance of the MMIPs for the adsorption of rutin in the analysis of Chinese medicinal plants was assessed. The mean recoveries were 84.33% for Saururus Chinensis (Lour.) Bail and 85.20% for Flos Sophorae, respectively, which showed that the prepared MMIPs with many advantages possess the value of the practical application. Kuhn et al. (2020) reported a new sample cleaning method based on the combination of micro matrix solid-phase dispersion (MSPD) and aqueous phase capacitive magnetic molecularly imprinted polymer (MMIP) for the determination of melamine in emulsion samples. Under optimized conditions, the proposed method exhibited a wide linear response in the concentration range of 250.0–5000.0 μg/L, satisfactory recoveries for all samples, and supreme repeatability (RSD <6.3%) were achieved as well. The low limit of detection close to 67.0 μg/L implied high ability of the proposed method for isolation of melamine from complicated milk samples. Gao et al. (2019) prepared core-shell magnetic molecularly imprinted polymer nanoparticles (MMIPs) for the adsorption and the isolation of tetracycline from milk samples. The results show that MMIPs have high adsorption capacity and good selectivity, and can be separated directly by magnetic separation.

## Application of MIPs

**Figure 10:**
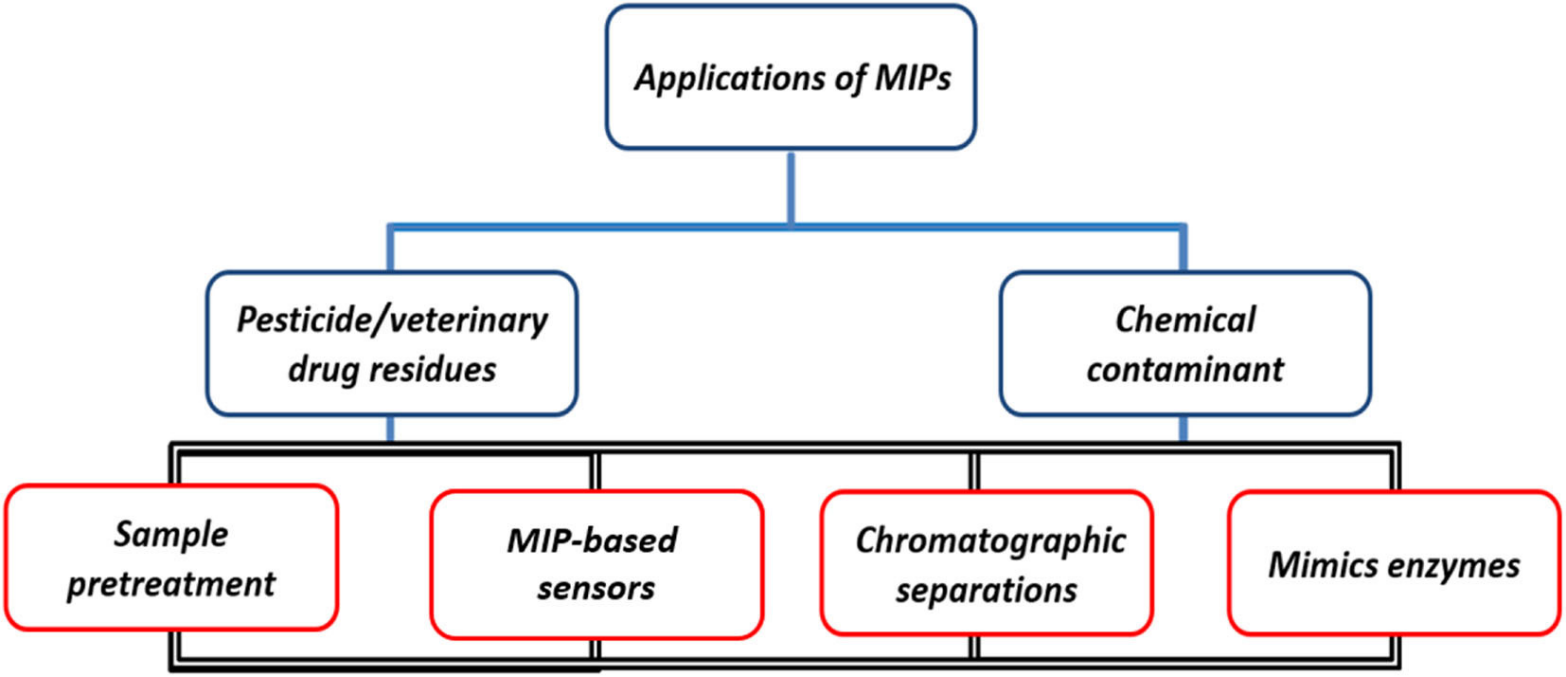
Application of MIPs in various fields.

As a novel polymeric material, MIPs exhibit a high binding capacity to target molecules and are characterized by unique recognition, good selectivity, and stable adsorption (Chen et al., 2016). Especially in the past decade, due to the complexity of detection samples, the diversity of chemical contaminants, and low residue limits, it is difficult to achieve high-throughput and rapid detection of trace chemical contaminant samples in food, and many efforts have been made in the field of MIPs research to improve the accuracy and sensitivity of food detection technology (Chiou et al., 2015; Chiesa et al., 2016; Jahanban-Esfahlan et al., 2020). The development of novel MIPs based on traditional MIPs has been greatly facilitated by designing suitable functional templates, synthesizing special functional monomers and cross-linkers, optimizing binding sites, and improving conditions. Today, MIPs are used not only for the separation or detection of many compounds in different applications, but also for catalysis and organic synthesis (Han et al., 2016; Lucci et al., 2017; Bagheri et al., 2018; Zhang et al., 2019). Figure 10 shows that MIPs have a wide range of applications in sample pre-treatment (SPE), chemomimetic sensing, chromatographic separation, and enzyme simulation. In this section, we provide a comprehensive summary and discussion of representative applications of MIPs in the analysis and detection of food samples.

### Sample Pretreatment

Sample pretreatment helps to eliminate matrix interference and to extract and enrich trace targets. However, conventional pretreatment can have time-consuming, tedious steps, and high reagent consumption (Płotka-Wasylka et al., 2016). Therefore, there is an urgent need to develop pretreatments with high selectivity and simple, time-saving, and labor-saving operations. Because MIPs have high specific recognition and selective adsorption for template molecules that can be subsequently eluted, they can be used as solid-phase extractants for specific enrichment and separations. The low cost, simple operation and high extraction efficiency stimulate wide use for sample pretreatment (Pataer et al., 2019). The comparison of different detection methods for chemical pollutants based on Sample pretreatment is given in Table 1.

**Table 1:** Comparison of different detection methods for chemical pollutants based on sample pretreatment.

**MIPs**	**Analytes**	**Samples**	**Analytical technique**	**LOD**	**Qmax**	**References**
MIPs/Fe_3_O_4_-C_3_N_4_	Atrazine	Water	HPLC	–	0.392 mg/g	Liu et al., 2015
MIPs/SiO_2_	Melamine	Milk	HPLC	2.5 mg/L	7.719 mg/g	Cheng et al., 2013
MIPs/MWNTs-SiO_2_	1, 4-dihydroxyanthraquinone	Water	UV-Vis	–	19.902 mg/g	Zhao et al., 2017
MIPs/Borosilicate glass	Sulphonylurea herbicides	Rice field water	HPLC	10.1–50.0 ng/L	1.15 mg/g	Tang et al., 2014
MIPs/Fe_3_O_4_@SiO_2_@C=C	Neonicotinoid insecticide Paichongding	Water	UV-Vis	17.30 mg/g	–	Zhang et al., 2016
Fe_3_O_4_@SiO_2_-MIPs	Melatonin	Portulaca oleracea	HPLC/UV	0.046 ng/mL	–	Dil et al., 2020
Double-template/MIP_S_	Fluoroquinolones/sulfonamides	Pork/chicken meat	HPLC	1.0–3.4 ng/g.	–	Song et al., 2017
DMIPs	Bisphenol A (BPA)	Sewage	HPLC	0.0007–16.3 ng/L	–	Sun et al., 2018
	Tetrabromobisphenol A	Sludge	HPLC	0–8.28 ng/g.	–	
H-MIPs	Enrofloxacin	Fish	HPLC	0.24 ng/mL	–	Tang et al., 2015
DMIPs	Amitraz	Urine	HPLC	0.56 ng/mL	–	Gholivand et al., 2015
MIPS-film	Triazine	Grain/vegetables	HPLC	0.04–0.12 μg/L	–	Hu et al., 2007
Atrazine-MIPs	Triazine	Soil	HPLC	–	–	Xu et al., 2011

#### Solid-Phase Extraction (SPE)

SPE based on MIPs, or MISPE, has been widely used in various solid phase extraction modes due to the structural predictability of MIPs, which allows them to form composites with various properties with other materials. Researchers have also conducted extensive experiments to synthesize better MIPs and have innovated the SPE method toward fewer steps, simplicity, economy, automation, miniaturization, time saving, and environmental friendliness and improved advantages (Arabi et al., 2020b; Büyüktiryaki et al., 2020; Ghorbani et al., 2020; Háková et al., 2020).

To date, more and more researchers have prepared different kinds of MIPs as adsorbents in extraction technology to obtain higher extraction recovery. The method of combining MIPs with SPE is of great significance to identify and detect the residues of agricultural veterinary drugs in real samples (Zhang et al., 2016). For example. Arias et al. (2020) synthesized a selective MIP-SPE sorbent for the extraction of chlorpyrifos, diazinon, and their corresponding oxygen radical forms in water by ontology polymerization and performed HPLC-UV analysis. The results showed that the MIP prepared using diazinon as a template molecule had the best recognition ability and significant affinity for these compounds. The potential of MIP was demonstrated by its application in different types of environmental water samples. This method also provides satisfactory detection limits using common equipment that is available to most analytical laboratories. Therefore, it is a promising alternative for monitoring the detection of chlorpyrifos, diazinon, and their oxo forms. Recently, Moreno-Gonzalez et al. (2020) reported on an online MISPE-CZE-MS/MS assay for the determination of patulin in apple foods. A preconcentration factor of 1,200 was achieved using this strategy compared to conventional hydrodynamic injection. This method not only improves the sensitivity of capillary electrophoresis, but also increases laboratory throughput, especially in terms of automation and precision, which is a breakthrough. Finally, this method was applied to the determination of patulin in apple beverages without interference from 5-HMF, with very satisfactory results.

#### Solid-Phase Microextraction (SPME)

Since its introduction by Arthur and Pawliszyn (1990) in 1990, solid phase microextraction (SPME) has been widely used for the analysis and detection of trace compounds in complex matrices due to its simplicity of operation, high extraction speed, low solvent consumption, high net extraction efficiency, and good compatibility (Jian et al., 2020; Khan et al., 2020). The quantitative but incomplete transfer of analytes on an equilibrium basis, whose own efficiency efficiency depends largely on the nature of the adsorbent material, makes the use of MIPs as SPME coatings a good solution, unlike SPEs, where the volume of the SPME adsorbent is usually much smaller and the material is usually coated on the surface of fused silica fibers. With the continued exploration of molecularly imprinted polymers, it has been possible to serve well in the field of food detection technology (Mirzajani et al., 2020).

In one example, Alipanahpour Dil et al. (2020) successfully synthesized Magnetic dual-template molecularly imprinted polymer (Fe_3_O_4_@SiO_2_-MDMIP) with high selectivity for p-Coumaric acid (p-CA) and ferulic acid (FA), and the obtained Fe_3_O_4_@SiO_2_-MDMIP with uniform particle size and strong selective adsorption capacity were suitable for the SS-MSPME process, which avoided the defect of template leakage during sample pretreatment. Meanwhile, the established SS-MSPME coupled with HPLC-UV had good recovery, reproducibility, and high sensitivity for both p-CA and FA in pomegranate, grape, and orange samples. The chromatographic peaks of the interfering substances were significantly different from the p-CA and FA peaks and had good discrimination ability for p-CA and FA. Recently, Mirzajani et al. (2020) prepared hollow fibers and monolithic fibers using metal-organic backbone deep eutectic solvent/molecularly imprinted polymers (MOF-DES/MIPs) and performed microextraction detection of phthalates using hollow fiber liquid film protected solid phase microextraction (HFLMP-SPME) and gas chromatography-flame ionization, compared to conventional metal-organic backbone/molecularly imprinted polymers. MOF-DES/MIPs monomer fibers have the following characteristics: high capacity for adsorption and desorption of analytes on the polymer surface, fast adsorption and desorption kinetics, improved analyte localization targets, and increased incorporation rates of target compounds in polymer tissues. Subsequently, the method was successfully applied to the determination of phthalates in yogurt, water, and soybean oil samples with satisfactory results. The method has much lower detection limits than other established phthalate assays, mainly due to the two-stage pre-enrichment resulting from the combination of both liquid-phase microextraction and solid-phase microextraction.

### MIP-Based Sensors

MIPs have been used as recognition units in sensors that specifically bind to target molecules and output detection signals. MIPs are simple, short preparation processes, physicochemically stable, and specific. Therefore, it is feasible to use MIPs instead of antibodies as sensitive recognition units (Abdollahi et al., 2018). Recent studies have shown that MIP-based sensors successfully combine the advantages of MIP receptors and various sensing platforms, and have great potential for application in food detection. MIPs devices based on their signal transduction principles include optical (Liu G. et al., 2018), electrochemical (Ai et al., 2018), and biosensing technologies (Zhang et al., 2018). The comparison of different detection methods for chemical pollutants based on MIPs-Sensor is given in Table 2. Some of the applications of different MIP-based sensors in food contaminant detection in the last 5 years are discussed below.

**Table 2:** Comparison of different detection methods for chemical pollutants based on MIPs-Sensor.

**MIPs**	**Analytes**	**Samples**	**Analytical technique**	**LOD**	**Qmax**	**References**
MIPs/MOFs	Methomyl pesticide	Pear	Electrochemical	0.0689 mg/L	3.217 mg/g	Qian et al., 2016
MIPs/AuNPs	Ractopamine	Swine feed	QCM	1.17 μmol/L	–	Kong et al., 2014
MIPs-GO/GCE	Thiamethoxam	Grain	Electrochemical	0.04 μmol/L	–	Xie et al., 2017
MIPs/Fe_3_O_4_@SiO_2_	Melamine	Milk	Fluorescence	0.05 mg/L	0.853 mg/g	Liu X. et al., 2018
MIPs/SiO_2_-FITC	Cyhalothrin	Chinese spirits	Fluorescence	9.17 nmol/L	–	Wang et al., 2015
AuNCs@SiO_2_@MIPs	Bovine serum albumin (BPA)	Seawater	Fluorescence	–	–	Wu et al., 2015
CQD-MIPs	Promethazine hydrochloride	Human plasma	Fluorescence	0.5 μmol/L	–	Ensafi et al., 2018
MIPs/Fe_3_O_4_-chitosan	Atrazine	Environment water	Fluorescence	0.86 μmol/L	0.709 mg/g	Liu et al., 2016
MIPs/AuNCs@SiO_2_	Bisphenol A	Seawater	Fluorescence	0.1 μmol/L	–	Wu et al., 2015
AuNPs@MIPs	Aflatoxins	Peanut/Corn	SPR	1.04 pg/mL	–	Akgonullu et al., 2020
CDs@MIPs	Sterigmatocystin	Cereals	Fluorescence	–	–	Xu et al., 2016
CDs@MIPs	Cyhalothrin	Water	Fluorescence	9.17 nmol/L	–	Wang et al., 2015

#### MIP-Based Electrochemical Sensors

Today, MIPs can be used as the most critical recognition probes for the development of this sensor, but molecularly imprinted polymers as adapters for antibody substitutes also still belong to biometric components, which have high cost and poor stability, which also makes electrochemical sensors have shortcomings in terms of selectivity. Electrochemical materials immobilized on the electrode surface allow the preparation of solid-state electrodes, which will help reduce expensive reagent consumption, reduce costs, simplify the sensor assembly, and improve stability, reproducibility, and signal enhancement (Cui et al., 2020; Gonçalves, 2020; Zhao et al., 2020).

Zhang et al. (2020) developed a highly selective molecularly imprinted electrochemiluminescence (MIECL) sensor for the determination of bisphenol A (BPA) based on molecularly imprinted Fe_3_O_4_ nanocrystals (MIP-Fe_3_O_4_/NCs) and luminescence. The synthesized MIP-Fe_3_O_4_-NCs were immobilized on the surface of glassy carbon electrode (GCE) to prepare the electrochemiluminescence (ECL) system. The GCE modified with MIP-Fe_3_O_4_-NCs can significantly enhance the cathodic ECL of luminol. After incubation in BPA solution, the imprinted sites on the surface of MIP-Fe_3_O_4_-NCs can specifically re-bind BPA and achieve selective and sensitive ECL bursting by hindering the electron transfer ability. The designed MIECL sensor has good selective, high sensitivity, and good accuracy and precision. Finally, the MIECL sensor was used for the sensitive and selective detection of BPA in fish and seawater samples, and the developed MIECL sensor was validated to have good analytical performance and has a broad application in the sensitive detection of BPA residues in aquaculture samples. Similarly, A graphene/molecularly imprinted electrochemical sensor constructed by Xie et al. (2017) was used to identify thiamethoxam pesticide residues in cereals. To avoid imprinted films that were too thick, vinyl benzoic acid was used as a functional monomer to be placed as an ultra-thin imprinted film on the surface of graphene. The sensor exhibited thiamethoxam recognition with an imprinting factor of 2.36, a concentration range of 0.5–20 μmol/L, and a detection limit of 0.04 μmol/L. For imidacloprid detection, Kong's group (Kong et al., 2013) prepared an electrochemically imprinted membrane on a glassy carbon electrode modified with reduced graphene oxide by electropolymerization of poly (o-phenylenediamine). The linear imidacloprid detection range was 0.75–70.00 μmol/L, and the detection limit was 0.40 μmol/L.

#### MIP-Based Optical Sensors

MIP-based optical sensors have been one of the most preferred technologies by researchers, which has greatly broadened the field of sensors for MIPs due to the simplicity of preparation, low achievable detection limits, and good visualization of expected effects. Nowadays, with the rapid development of nanomaterial types, materials such as quantum dots (Bhogal et al., 2020), magnetic nanoparticles (Chepyala, 2020), nanosilver/gold (Mahmoudpour et al., 2020), metal-organic frameworks (Svitkova and Palchetti, 2020), and graphene oxide (Pandey et al., 2020) exhibit their unique optical properties of narrow emission and resistance to fluorescence bursting, which have great potential for fluorescent sensors, probes and labels. For instance: Cao et al. (2020) established a sensitive fluorescence sensor for the detection of octopamine (OA). The dispersion and emission intensity in the organic phase can be significantly improved by encapsulating upconversion nanoparticles (UCNPs) in a metal-organic skeleton (ZIF-8) in combination with a molecularly imprinted polymer (MIP). At the same time, the ZIF-8 membrane reduces the mass transfer resistance and adsorption time of the molecularly imprinted polymer. The sensor at UCNPs@ZIF-8@MIP has excellent optical properties similar to those of natural receptors and superior selective discrimination. Compared with traditional detection methods, this method has a detection limit of 0.081 mg/L, which can achieve both specific recognition and quantitative detection of OA without complex pre-processing, and is promising for practical applications in the detection of OA in complex food matrices.

**Figure 11:**
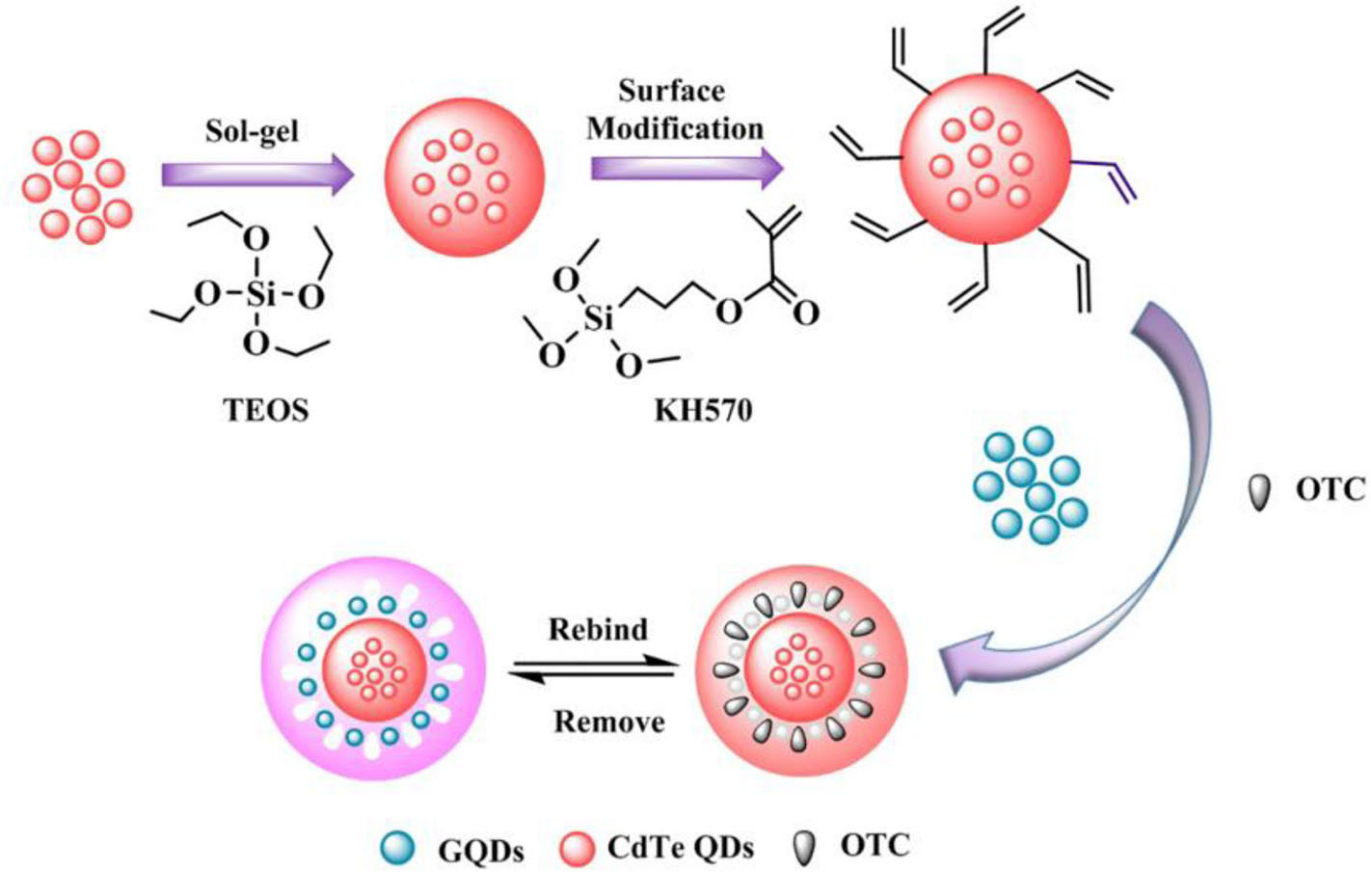
The synthesis process of GQDs/CdTe@MIPs (Wang et al., 2020). Reprinted with permission from Elsevier. Copyright (2020) Elsevier.

Recently, Wang et al. (2020) proposed a proportional fluorescence imprinting sensors (GQDs/CdTe@MIPs) to successfully construct a method for the identification and detection of oxytetracycline (OTC) in milk samples using precipitation polymerization, and the synthesis is shown in Figure 11. GQDs/CdTe@MIPs were established on the basis of precipitation polymerization with GQDs as the response signal and CdTe quantum dots as the reference signal, so that the GQDs/ The CdTe@MIPs have strong fluorescence stability and good sensitivity for OTC recognition. At the same time, due to the existence of corresponding recognition sites, the fluorescence color of GQDs/CdTe@MIPs changed significantly from blue to pink as the OTC concentration increased, enabling the visual detection of OTC.

### Chromatographic Separations

The stationary phase using MIPs as molecularly imprinted separations is one of the most important components in the field of chromatographic separation research. In fact, it can be established and applied to sample pre-processing not only by “pre-concentrating” the analyte and eliminating matrix interference to lower the detection limit of the method, but also by removing interferents and/or pre-concentrating and/or deriving the analyte into a chemical more suitable for detection (Lobato et al., 2020). Where the sample matrix is complex and heavily contaminated, molecularly imprinted polymers can be used to achieve the appropriate selectivity and sensitivity. Currently, MIP techniques are combined with many different instrumentation and/or detection techniques to obtain optimal analytical parameters, including capillary electrophoresis (CE), gas chromatography (GS), and high performance liquid chromatography (HPLC), and the separation in combination with MIPs provides more pronounced selectivity, high affinity, and rapid predictability compared to conventional chromatographic separation phases. This section focuses on the application of MIPs in the field of chromatographic separation in the last 3 years.

**Figure 12:**
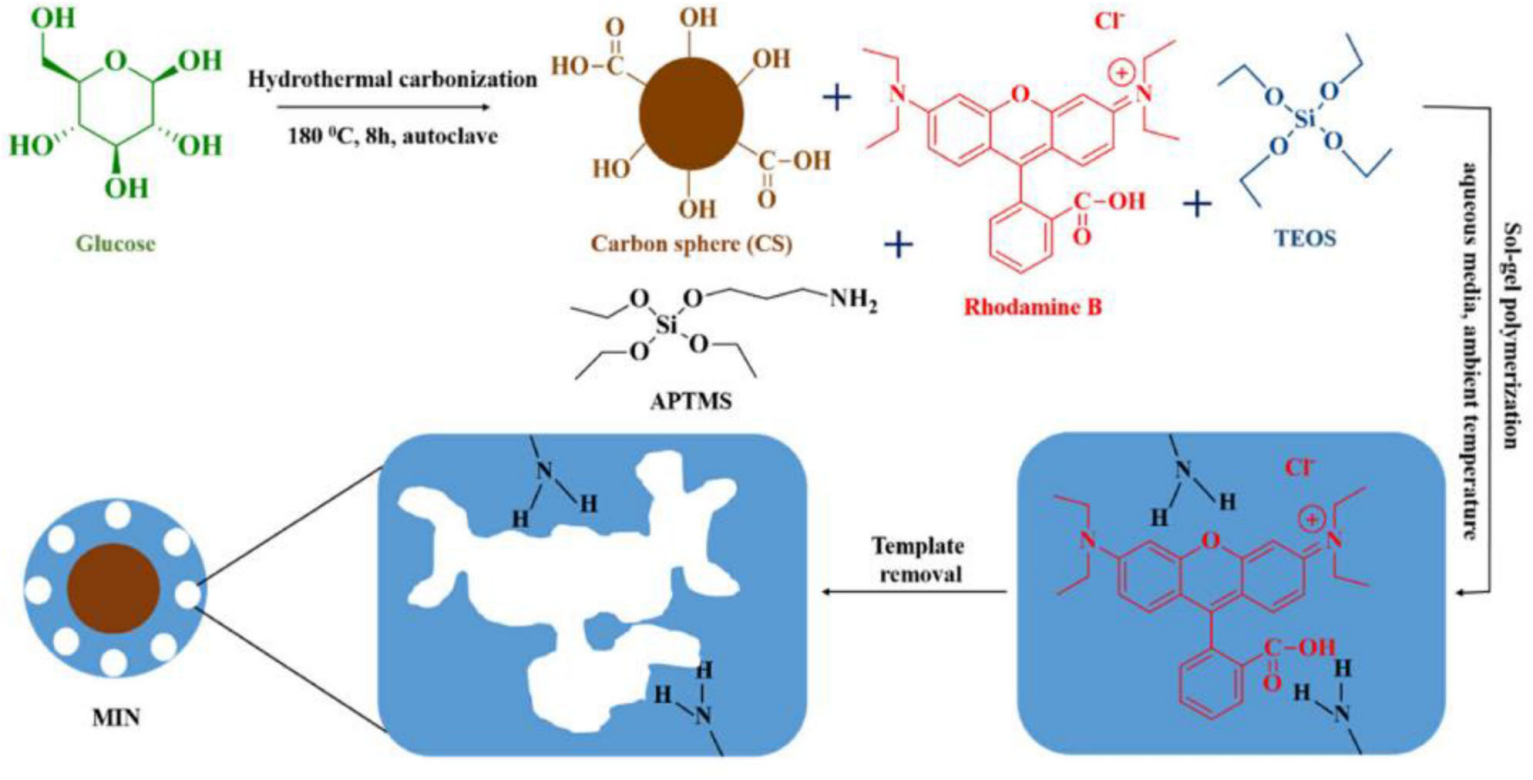
Schematic illustration for the basic preparation procedure of MINs (Arabi et al., 2020a). Reprinted with permission from Elsevier. Copyright (2020) Elsevier.

Rapid chromatographic separation technology, as an important means of organic concentration and sample purification, has the characteristics of large loading rate, good separation effect, and wide application range. For instance. Gao et al. (2015) synthesized Sudan I imprinted polymers by employing the interaction between Sudan I (template) and methacrylic acid (functional monomer), followed by washing to remove Sudan I leaving the Sudan I-binding sites exposed. MIPs were used as a stationary phase for TLC and could selectively retain Sudan I at the original spot with little interference. Yu et al. (2019) prepared a highly selective molecularly imprinted polymer (MIP) for extraction and preconcentration of salidroside using salidroside (SD) as a template, acrylamide (AM) as a functional monomer. Under the optimum conditions, a rapid, economical, and efficient method based upon MIP-SPE coupled with high-performance liquid chromatography (HPLC) was developed for the determination of SD in Rhodiola crenulata. The method showed satisfactory recoveries of 88.74–97.64% with relative standard deviations (RSDs) ranging from 2.05–3.54%. Recently, a simple, sensitive and reliable method for the determination of RhB in food by high performance liquid chromatography based on MINs has been reported. Arabi et al. (2020a) synthesized hydrophilic molecularly imprinted nanospheres (MINs) using surface imprinting technique and applied them as dispersant adsorbents in matrix solid phase dispersion (MSPD) system, which was successfully applied for the direct selective extraction of RhB from solid and semi-solid food samples (Figure 12). This method utilizes the synergistic interaction of Carbon sphere (CS) and sol-gel precursors in aqueous media to rationally prepare low-cost hydrophilic MINs on the basis of surface imprinting.The design of the MINs allows the synthesis conditions to be fully compatible with the principles of green chemistry and the resulting materials are environmentally friendly. Thus, a straightforward, economical and non-toxic preparation method allows the large-scale production of MINs with great potential for commercialization.

### Mimics Enzymes

Enzymes catalyze biochemical reactions *in vivo* and *in vitro* with high efficiency and specificity. However, natural enzymes have poor stability and resistance, a low reuse rate; they are difficult to prepare and extract, difficult to preserve and transport and the catalytic reactions require appropriate pH and temperature. These issues have severely restricted natural enzymes in biochemical applications, agricultural production, food manufacturing, and analysis (Wang et al., 2016). MIPs have unique advantages in replacing natural enzymes. Molecular imprinting of recognition sites and reactive groups of enzyme active centers in the interior of polymers were used to obtain molecularly imprinted enzymes (Daoud Attieh et al., 2017) with catalytic activity.

**Figure 13:**
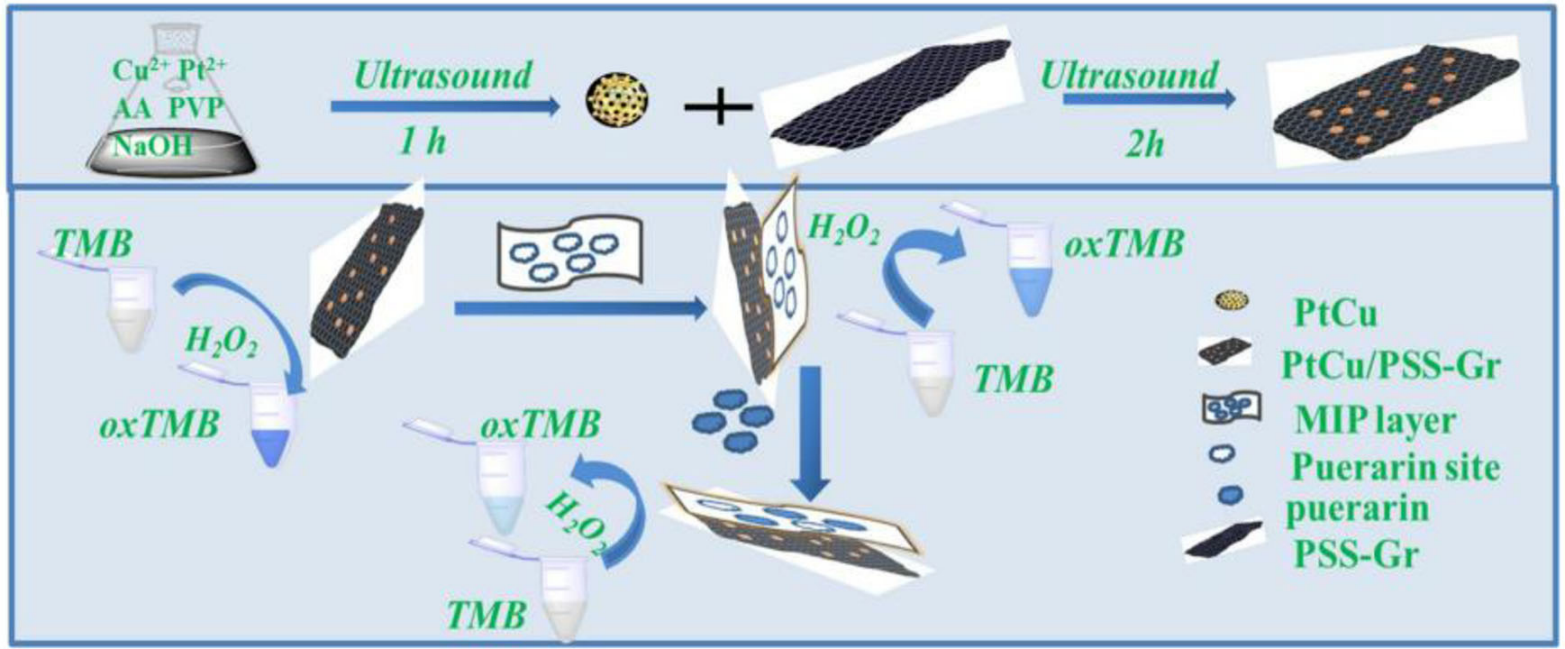
Schematic diagram for the preparation of MIP@PtCu/PSS-Gr nanocomposite to detect puerarin (Guo et al., 2020). Reprinted with permission from Elsevier. Copyright (2020) Elsevier.

In 1987, Mosbach's group (Leonhardt and Mosbach, 1987) first synthesized molecularly imprinted enzyme mimics and applied them to the hydrolysis of p-nitrophenyl acetate. Subsequently, Liu and Wulff (2008) prepared MIPs with the catalytic activity of carboxyphthalase A by using transition state analogs as templates. The imprinted polymers rapidly catalyzed the hydrolysis of carbonic acid. Through the imprinting synthesis of substrate or transition state analogs of enzymatic reactions, catalytic and biochemical reaction mechanisms can be studied, and the enzymatic reaction can be controlled. Molecularly imprinted biomimetic catalysis resembles chemical catalysis, which simulates the principle of antigen-antibody interactions. It combines the characteristics of chemical catalysts and biocatalysts and has the advantages of more specific catalysis, mild reaction conditions, and high efficiency (Yuan et al., 2018a). For instance, Bagheri et al. (2018) prepared a novel molecularly imprinted enzyme mimic with catalase-like activity by depositing a silicon molecularly imprinted layer on the surface of AgNPs@ZnMOF. The imprinted enzyme could mimic catalase oxidation of terephthalic acid to produce fluorescent molecules. When the active site on the surface of the molecularly imprinted enzyme was bound to the patulin molecule, it prevented hydrogen peroxide and terephthalic acid from contacting AgNPs@ZnMOF inside the enzyme. Catalytic oxidation reactions were blocked, and no fluorescence was emitted. A fluorescence detection method for patulin based on the molecularly imprinted enzyme mimic was thus established, with a detection limit of 0.06 μmol/L. Also besides, Guo et al. (2020) combined PtCu/PSS-Gr nanocomposites formed by deposition of PtCu bimetallic nanoparticles on polystyrene sulfonate (PSS) functionalized graphene (Gr) with molecularly imprinted polymer (MIP) to propose a new colorimetric assay for puerarin and apply it to the study of enzymatic activity (Figure 13). The combination of MIP with PtCu/PSS-Gr nanocomposite monomers under the synergistic effect of PtCu/PSS-Gr nanocomposites not only makes the determination of puerarin highly selective due to the large specific surface area of PSS-Gr, good dispersion, strong adsorption capacity to substrates and strong peroxidase activity, but also utilizes the catalytic activity of the nanoenzymes similar to peroxidase. The detection of small molecules that are neither substrates for nanoenzymes nor highly redox-active substances has been achieved, expanding the field of analytical applications.

## Conclusions and Perspectives

The development of molecularly imprinted polymers (MIPs) has made considerable progress, and nanoscale MIPs, with remarkable binding properties and selectivity compared to conventional imprinted molecular polymers, can be used as solid-phase extraction materials for the separation and enrichment of chemical contaminants in sample pretreatment processes. In addition, MIPs are outstandingly resistant to high temperature and pressure, acid and alkali, recyclable, and easy to store, making them suitable as sensitive materials for sensors for the analytical detection of real samples. To date, MIPs have been developed from single templates to composite templates, and the preparation process has been continuously optimized to improve the application range, adsorption performance and specific selectivity. They have been widely used in various fields such as environmental pollutant analysis, food quality and safety, and biological sample separation and enrichment. However, there are still some problems to be explored and solved.

(1) MIPs usually show the best performance in hydrophobic organic solvents, which leads to the presence of polar solvents (especially water) in practical applications of MIPs that can seriously interfere with the formation of pre-polymerized complexes in the imprinting process and disrupt the interaction between the monomer and the template, which can be used in the future.

(2) The excellent performance of nanostructured MIP materials lies not only in their size, but also in their rapid equilibrium with the substance to be measured, but due to the difficulty of removing the template completely after the preparation stage of the highly cross-linked polymer (template molecule), MIP materials often suffer from problems such as template leakage, resulting in the preparation of molecularly imprinted nanomaterials with irregular particle shapes, different particle sizes, non-uniform recognition sites and low affinity.

(3) In the synthesis of molecularly imprinted polymers, there are limited varieties of functional monomers and cross-linking agents available, and the chemical reagents used are not only toxic, but also face problems such as high capital expenditure and low conversion efficiency, making it difficult to achieve mass production from laboratory to factory and unable to maximize commercial conversion.

Efforts to solve these problems never cease, and in the future, the combination of MIP with other porous or nanostructured materials may provide new approaches to develop chemical contaminants for use in food, and in particular, MIP/porous polymers and carbon nanomaterials will be a major breakthrough in the field of biotech sensors. In addition, the use of MIP in combination with different analytical instruments to artificialize detection systems is also an ideal goal that is constantly being pursued and may be realized in the near future.

## Author Contributions

GL and DX conceived and designed this review. MG wrote the paper. YG, GC, JL, XX, and XH revised the manuscript.

## Conflict of Interest

The authors declare that the research was conducted in the absence of any commercial or financial relationships that could be construed as a potential conflict of interest.

## References

[B1] Abdollahi E. Khalafi-Nezhad A. Mohammadi A. Abdouss M. Salami-Kalajahi M. (2018). Smynthesis of new molecularly imprinted polymer via reversible addition fragmentation transfer polymerization as a drug delivery system. Polymer 143, 245–257. 10.1016/j.polymer.2018.03.058

[B2] Ai J. Guo H. Xue R. Wang X. Lei X. Yang W. (2018). A self-probing, gate-controlled, molecularly imprinted electrochemical sensor for ultrasensitive determination of p-nonylphenol. Electrochem. Commun. 89, 1–5. 10.1016/j.elecom.2018.02.008

[B3] Akgonullu S. Yavuz H. Denizli A. (2020). SPR nanosensor based on molecularly imprinted polymer film with gold nanoparticles for sensitive detection of aflatoxin B1. Talanta 219:121219. 10.1016/j.talanta.2020.121219 32887120

[B4] Akhoundian M. Alizadeh T. Ganjali M. R. Norouzi P. (2019). Ultra-trace detection of methamphetamine in biological samples using FFT-square wave voltammetry and nano-sized imprinted polymer/MWCNTs -modified electrode. Talanta 200, 115–123. 10.1016/j.talanta.2019.02.027 31036164

[B5] Alipanahpour Dil E Ghaedi M. Asfaram A. Mehrabi F. Shokrollahi A. Matin A. A.. (2020). Magnetic dual-template molecularly imprinted polymer based on syringe-to-syringe magnetic solid-phase microextraction for selective enrichment of p-Coumaric acid and ferulic acid from pomegranate, grape, and orange samples. Food Chem. 325:126902. 10.1016/j.foodchem.2020.126902 32387937

[B6] Andersson L. I. Paprica A. Arvidsson T. (1997). A highly selective solid phase extraction sorbent for pre-concentration of sameridine made by molecular imprinting. Chromatographia 46, 57–62. 10.1007/BF02490930

[B7] Ansari S. (2017). Combination of molecularly imprinted polymers and carbon nanomaterials as a versatile biosensing tool in sample analysis: recent applications and challenges. Trends Anal. Chem. 93, 134–151. 10.1016/j.trac.2017.05.015

[B8] Ansell R. J. Mosbach K. (1998). Magnetic molecularly imprinted polymer beads for drug radioligand binding assay. Analyst 123, 1611–1616. 10.1039/a801903g 9830174

[B9] Arabi M. Ghaedi M. Ostovan A. (2016a). Development of dummy molecularly imprinted based on functionalized silica nanoparticles for determination of acrylamide in processed food by matrix solid phase dispersion. Food Chem. 210, 78–84. 10.1016/j.foodchem.2016.04.080 27211623

[B10] Arabi M. Ostovan A. Bagheri A. R. Guo X. Li J. Ma J.. (2020a). Hydrophilic molecularly imprinted nanospheres for the extraction of rhodamine B followed by HPLC analysis: a green approach and hazardous waste elimination. Talanta 215:120933. 10.1016/j.talanta.2020.120933 32312469

[B11] Arabi M. Ostovan A. Bagheri A. R. Guo X. Wang L. Li J. (2020b). Strategies of molecular imprinting-based solid-phase extraction prior to chromatographic analysis. Trends Anal. Chem. 128:115923 10.1016/j.trac.2020.115923

[B12] Arabi M. Ostovan A. Ghaedi M. Purkait M. K. (2016b). Novel strategy for synthesis of magnetic dummy molecularly imprinted nanoparticles based on functionalized silica as an efficient sorbent for the determination of acrylamide in potato chips: optimization by experimental design methodology. Talanta 154, 526–532. 10.1016/j.talanta.2016.04.010 27154710

[B13] Arias P. G. Martinez-Perez-Cejuela H. Combes A. Pichon V. Pereira E. Herrero-Martinez J. M.. (2020). Selective solid-phase extraction of organophosphorus pesticides and their oxon-derivatives from water samples using molecularly imprinted polymer followed by high-performance liquid chromatography with UV detection. J. Chromatogr. A 1626:461346. 10.1016/j.chroma.2020.461346 32797826

[B14] Arthur C. L. Pawliszyn J. (1990). Solid phase microextraction with thermal desorption using fused silica optical fibers. Anal. Chem. 62, 2145–2148. 10.1021/ac00218a019

[B15] Ashley J. Shahbazi M.-A. Kant K. Chidambara V. A. Wolff A. Bang D. D.. (2017). Molecularly imprinted polymers for sample preparation and biosensing in food analysis: progress and perspectives. Biosens. Bioelectron. 91, 606–615. 10.1016/j.bios.2017.01.018 28103516

[B16] Azizi A. Bottaro C. S. (2020). A critical review of molecularly imprinted polymers for the analysis of organic pollutants in environmental water samples. J. Chromatogr. A 1614:460603. 10.1016/j.chroma.2019.460603 31629490

[B17] Bagheri A. R. Arabi M. Ghaedi M. Ostovan A. Wang X. Li J.. (2019). Dummy molecularly imprinted polymers based on a green synthesis strategy for magnetic solid-phase extraction of acrylamide in food samples. Talanta 195, 390–400. 10.1016/j.talanta.2018.11.065 30625559

[B18] Bagheri A. R. Ghaedi M. (2020). Green preparation of dual-template chitosan-based magnetic water-compatible molecularly imprinted biopolymer. Carbohydr. Polym. 236:116102. 10.1016/j.carbpol.2020.116102 32172901

[B19] Bagheri N. Khataee A. Habibi B. Hassanzadeh J. (2018). Mimetic Ag nanoparticle/Zn-based MOF nanocomposite (AgNPs@ZnMOF) capped with molecularly imprinted polymer for the selective detection of patulin. Talanta 179, 710–718. 10.1016/j.talanta.2017.12.009 29310298

[B20] Barsbay M. Güven O. (2018). Nanostructuring of polymers by controlling of ionizing radiation-induced free radical polymerization, copolymerization, grafting and crosslinking by RAFT mechanism. Radiat. Phys. Chem. 169:107816 10.1016/j.radphyschem.2018.04.009

[B21] BelBruno J. J. (2019). Molecularly imprinted polymers. Chem. Rev. 119, 94–119. 10.1021/acs.chemrev.8b00171 30246529

[B22] Beqqali A. E. Andersson L. I. Jeppsson A. D. Abdel-Rehim M. (2017). Molecularly imprinted polymer-sol-gel tablet toward micro-solid phase extraction: II. determination of amphetamine in human urine samples by liquid chromatography-tandem mass spectrometry. J. Chromatogr. B 1063, 130–135. 10.1016/j.jchromb.2017.08.027 28863334

[B23] Bhogal S. Kaur K. Malik A. K. Sonne C. Lee S. S. Kim K.-H. (2020). Core-shell structured molecularly imprinted materials for sensing applications. Trends Anal. Chem. 133:116043 10.1016/j.trac.2020.116043

[B24] Büyüktiryaki S. Keçili R. Hussain C. M. (2020). Functionalized nanomaterials in dispersive solid phase extraction: advances & prospects. Trends Anal. Chem. 127:115893 10.1016/j.trac.2020.115893

[B25] Canfarotta F. Rapini R. Piletsky S. (2018). Recent advances in electrochemical sensors based on chiral and nano-sized imprinted polymers. Curr. Opin. Electrochem. 7, 146–152. 10.1016/j.coelec.2017.11.018

[B26] Cao Y. Hu X. Zhao T. Mao Y. Fang G. Wang S. (2020). A core-shell molecularly imprinted optical sensor based on the upconversion nanoparticles decorated with Zinc-based metal-organic framework for selective and rapid detection of octopamine. Sens. Actuat. B Chem. 326:128838 10.1016/j.snb.2020.128838

[B27] Carter S. R. Rimmer S. (2004). Surface molecularly imprinted polymer core–shell particles. Adv. Funct. Mater. 14, 553–561. 10.1002/adfm.200305069

[B28] Carvalho F. P. (2017). Pesticides, environment, and food safety. Food Energy Sec. 6, 48–60. 10.1002/fes3.108

[B29] Chen L. Wang X. Lu W. Wu X. Li J. (2016). Molecular imprinting: perspectives and applications. Chem. Soc. Rev. 45, 2137–2211. 10.1039/C6CS00061D 26936282

[B30] Chen Y. Zhou S. Li L. Zhu J.-J. (2017). Nanomaterials-based sensitive electrochemiluminescence biosensing. Nano Today 12, 98–115. 10.1016/j.nantod.2016.12.013

[B31] Cheng W. Liu Z. Wang Y. (2013). Preparation and application of surface molecularly imprinted silica gel for selective extraction of melamine from milk samples. Talanta 116, 396–402. 10.1016/j.talanta.2013.05.067 24148421

[B32] Chepyala R. (2020). Applications and success of MIPs in optical-based nanosensors, in Nanofabrication for Smart Nanosensor Applications, eds Pal K. Gomes F. (Elsevier), 89–121. 10.1016/B978-0-12-820702-4.00004-0

[B33] Chiesa L. M. Labella G. F. Giorgi A. Panseri S. Pavlovic R. Bonacci S.. (2016). The occurrence of pesticides and persistent organic pollutants in Italian organic honeys from different productive areas in relation to potential environmental pollution. Chemosphere 154, 482–490. 10.1016/j.chemosphere.2016.04.004 27085062

[B34] Chiou J. Leung A. H. H. Lee H. W. Wong W.-T. (2015). Rapid testing methods for food contaminants and toxicants. J. Integr. Agric. 14, 2243–2264. 10.1016/S2095-3119(15)61119-4

[B35] Cui B. Liu P. Liu X. Liu S. Zhang Z. (2020). Molecularly imprinted polymers for electrochemical detection and analysis: progress and perspectives. J. Mater. Res. Technol. 9, 12568–12584. 10.1016/j.jmrt.2020.08.052

[B36] Dai H. Xiao D. He H. Li H. Yuan D. Zhang C. (2014). Synthesis and analytical applications of molecularly imprinted polymers on the surface of carbon nanotubes: a review. Microchim. Acta 182, 893–908. 10.1007/s00604-014-1376-5

[B37] Daoud Attieh M. Zhao Y. Elkak A. Falcimaigne-Cordin A. Haupt K. (2017). Enzyme-initiated free-radical polymerization of molecularly imprinted polymer nanogels on a solid phase with an immobilized radical source. Angew. Chem. Int. Ed. Engl. 56, 3339–3343. 10.1002/anie.201612667 28194847

[B38] Dil E. A. Doustimotlagh A. H. Javadian H. Asfaram A. Ghaedi M. (2020). Nano-sized FeO@SiO-molecular imprinted polymer as a sorbent for dispersive solid-phase microextraction of melatonin in the methanolic extract of, biological, and water samples. Talanta 221:121620. 10.1016/j.talanta.2020.121620 33076148

[B39] Du X.-W. Zhang Y.-X. She Y.-X. Liu G.-Y. Zhao F.-N. Wang J. (2016). Fluorescent competitive assay for melamine using dummy molecularly imprinted polymers as antibody mimics. J. Integr. Agric. 15, 1166–1177. 10.1016/S2095-3119(16)61357-6

[B40] Duan H. Li L. Wang X. Wang Y. Li J. Luo C. (2016). CdTe quantum dots@luminol as signal amplification system for chrysoidine with chemiluminescence-chitosan/graphene oxide-magnetite-molecularly imprinting sensor. Spectrochim. Acta A Mol. Biomol. Spectrosc. 153, 535–541. 10.1016/j.saa.2015.09.016 26433339

[B41] Ensafi A. A. Kazemifard N. Rezaei B. (2017). Development of a nano plastic antibody for determination of propranolol using CdTe quantum dots. Sens. Actuat. B Chem. 252, 846–853. 10.1016/j.snb.2017.06.078

[B42] Ensafi A. A. Nasr-Esfahani P. Rezaei B. (2018). Synthesis of molecularly imprinted polymer on carbon quantum dots as an optical sensor for selective fluorescent determination of promethazine hydrochloride. Sens. Actuat. B Chem. 257, 889–896. 10.1016/j.snb.2017.11.050

[B43] Erturk Bergdahl G. Andersson T. Allhorn M. Yngman S. Timm R. Lood R. (2019). *In vivo* detection and absolute quantification of a secreted bacterial factor from skin using molecularly imprinted polymers in a surface plasmon resonance biosensor for improved diagnostic abilities. ACS Sens. 4, 717–725. 10.1021/acssensors.8b01642 30758943

[B44] Figueiredo L. Erny G. L. Santos L. Alves A. (2016). Applications of molecularly imprinted polymers to the analysis and removal of personal care products: a review. Talanta 146, 754–765. 10.1016/j.talanta.2015.06.027 26695327

[B45] Gao F. Hu Y. Chen D. Li-Chan E. C. Y. Grant E. Lu X. (2015). Determination of Sudan I in paprika powder by molecularly imprinted polymers-thin layer chromatography-surface enhanced Raman spectroscopic biosensor. Talanta 143, 344–352. 10.1016/j.talanta.2015.05.003 26078169

[B46] Gao W. Li J. Li P. Huang Z. Cao Y. Liu X. (2019). Preparation of Magnetic Molecularly Imprinted Polymer (MMIP) Nanoparticles (NPs) for the selective extraction of tetracycline from milk. Anal. Lett. 53, 1097–1112. 10.1080/00032719.2019.1698049

[B47] Ghanbari F. Moradi M. (2017). Application of peroxymonosulfate and its activation methods for degradation of environmental organic pollutants: review. Chem. Eng. J. 310, 41–62. 10.1016/j.cej.2016.10.064

[B48] Gholivand M. B. Khodadadian M. Bahrami G. (2015). Molecularly imprinted polymer preconcentration and flow injection amperometric determination of 4-nitrophenol in water. Anal. Lett. 48, 2856–2869. 10.1080/00032719.2015.1060598

[B49] Ghorbani M. Aghamohammadhassan M. Ghorbani H. Zabihi A. (2020). Trends in sorbent development for dispersive micro-solid phase extraction. Microchem. J. 158:105250 10.1016/j.microc.2020.105250

[B50] Gonçalves L. M. (2020). Electropolymerized molecularly imprinted polymers (e-MIPs), perceptions based in recent literature for soon-to-be world-class scientists. Curr. Opin. Electrochem. 25:100640 10.1016/j.coelec.2020.09.007

[B51] Guo L. Zheng H. Zhang C. Qu L. Yu L. (2020). A novel molecularly imprinted sensor based on PtCu bimetallic nanoparticle deposited on PSS functionalized graphene with peroxidase-like activity for selective determination of puerarin. Talanta 210:120621. 10.1016/j.talanta.2019.120621 31987162

[B52] Guoning C. Hua S. Wang L. Qianqian H. Xia C. Hongge Z.. (2020). A surfactant-mediated sol-gel method for the preparation of molecularly imprinted polymers and its application in a biomimetic immunoassay for the detection of protein. J. Pharm. Biomed. Anal. 190:113511. 10.1016/j.jpba.2020.113511 32781321

[B53] Háková M. Havlíková L. C. Švec F. Solich P. Šatínský D. (2020). Nanofibers as advanced sorbents for on-line solid phase extraction in liquid chromatography: a tutorial. Anal. Chim. Acta 1121, 83–96. 10.1016/j.aca.2020.04.045 32493593

[B54] Han Q. Shen X. Zhu W. Zhu C. Zhou X. Jiang H. (2016). Magnetic sensing film based on Fe(3)O(4)@Au-GSH molecularly imprinted polymers for the electrochemical detection of estradiol. Biosens. Bioelectron. 79, 180–186. 10.1016/j.bios.2015.12.017 26706939

[B55] Haupt K. Mosbach K. (2000). Molecularly imprinted polymers and their use in biomimetic sensors. Chem. Rev. 100, 2495–2504. 10.1021/cr990099w 11749293

[B56] Hu X. Hu Y. Li G. (2007). Development of novel molecularly imprinted solid-phase microextraction fiber and its application for the determination of triazines in complicated samples coupled with high-performance liquid chromatography. J. Chromatogr. A 1147, 1–9. 10.1016/j.chroma.2007.02.037 17336991

[B57] Huang S. Xu J. Zheng J. Zhu F. Xie L. Ouyang G. (2018). Synthesis and application of magnetic molecularly imprinted polymers in sample preparation. Anal. Bioanal. Chem. 410, 3991–4014. 10.1007/s00216-018-1013-y 29651522

[B58] Huang X. Liu X. Luo Q. Liu J. Shen J. (2011). Artificial selenoenzymes: designed and redesigned. Chem. Soc. Rev. 40, 1171–1184. 10.1039/C0CS00046A 21125082

[B59] Im K. Nguyen D. N. Kim S. Kong H. J. Kim Y. Park C. S.. (2017). Graphene-embedded hydrogel nanofibers for detection and removal of aqueous-phase dyes. ACS Appl. Mater. Interfaces 9, 10768–10776. 10.1021/acsami.7b01163 28301130

[B60] Jafari M. T. Rezaei B. Zaker B. (2009). Ion Mobility spectrometry as a detector for molecular imprinted polymer separation and metronidazole determination in pharmaceutical and human serum samples. Anal. Chem. 81, 3585–3591. 10.1021/ac802557t 19361231

[B61] Jahanban-Esfahlan A. Roufegarinejad L. Jahanban-Esfahlan R. Tabibiazar M. Amarowicz R. (2020). Latest developments in the detection and separation of bovine serum albumin using molecularly imprinted polymers. Talanta 207:120317. 10.1016/j.talanta.2019.120317 31594596

[B62] Jian Y. Chen L. Cheng J. Huang X. Yan L. Li H. (2020). Molecularly imprinted polymers immobilized on graphene oxide film for monolithic fiber solid phase microextraction and ultrasensitive determination of triphenyl phosphate. Anal. Chim. Acta 1133, 1–10. 10.1016/j.aca.2020.08.003 32993861

[B63] Jiang S. Saito M. Murahashi M. Tamiya E. (2017). Pressure free nanoimprinting lithography using ladder-type HSQ material for LSPR biosensor chip. Sens. Actuat. B Chem. 242, 47–55. 10.1016/j.snb.2016.11.030

[B64] Khan W. A. Arain M. B. Soylak M. (2020). Nanomaterials-based solid phase extraction and solid phase microextraction for heavy metals food toxicity. Food Chem. Toxicol. 145:111704. 10.1016/j.fct.2020.111704 32853698

[B65] Kong L. Jiang X. Zeng Y. Zhou T. Shi G. (2013). Molecularly imprinted sensor based on electropolmerized poly(o-phenylenediamine) membranes at reduced graphene oxide modified electrode for imidacloprid determination. Sens. Actuat. B Chem. 185, 424–431. 10.1016/j.snb.2013.05.033

[B66] Kong L. J. Pan M. F. Fang G. Z. He X. L. Yang Y. K. Dai J.. (2014). Molecularly imprinted quartz crystal microbalance sensor based on poly(o-aminothiophenol) membrane and Au nanoparticles for ractopamine determination. Biosens. Bioelectron. 51, 286–292. 10.1016/j.bios.2013.07.043 23974160

[B67] Kuhn J. Aylaz G. Sari E. Marco M. Yiu H. H. P. Duman M. (2020). Selective binding of antibiotics using magnetic molecular imprint polymer (MMIP) networks prepared from vinyl-functionalized magnetic nanoparticles. J. Hazard. Mater. 387:121709. 10.1016/j.jhazmat.2019.121709 31812475

[B68] Kupai J. Razali M. Buyuktiryaki S. Kecili R. Szekely G. (2017). Long-term stability and reusability of molecularly imprinted polymers. Polym. Chem. 8, 666–673. 10.1039/C6PY01853J 28496524 PMC5361172

[B69] Leonhardt A. Mosbach K. (1987). Enzyme-mimicking polymers exhibiting specific substrate binding and catalytic functions. React. Polym. Ion Exchang. Sorb. 6, 285–290. 10.1016/0167-6989(87)90099-7

[B70] Li M. Li R. Tan J. Jiang Z. T. (2013). Titania-based molecularly imprinted polymer for sulfonic acid dyes prepared by sol–gel method. Talanta 107, 203–210. 10.1016/j.talanta.2013.01.014 23598213

[B71] Li Y. Yang H.-H. You Q.-H. Zhuang Z.-X. Wan X.-R. (2006). Protein recognition via surface molecularly imprinted polymer nanowires. Anal. Chem. 78, 317–320. 10.1021/ac050802i 16383343

[B72] Liu G. Li T. Yang X. She Y. Wang M. Wang J.. (2016). Competitive fluorescence assay for specific recognition of atrazine by magnetic molecularly imprinted polymer based on Fe_3_O_4_-chitosan. Carbohydr. Polym. 137, 75–81. 10.1016/j.carbpol.2015.10.062 26686107

[B73] Liu G. She Y. Hong S. Wang J. Xu D. (2018). Development of ELISA-like fluorescence assay for melamine detection based on magnetic dummy molecularly imprinted polymers. Appl. Sci. 8:560 10.3390/app8040560

[B74] Liu G. Yang X. Li T. She Y. Wang S. Wang J. (2015). Preparation of a magnetic molecularly imprinted polymer using g-C_3_N_4_-Fe_3_O_4_ for atrazine adsorption. Mater. Lett. 160, 472–475. 10.1016/j.matlet.2015.07.157

[B75] Liu J.-Q. Wulff G. (2008). Functional mimicry of carboxypeptidase a by a combination of transition state stabilization and a defined orientation of catalytic moieties in molecularly imprinted polymers. J. Am. Chem. Soc. 130, 8044–8054. 10.1021/ja8012648 18510322

[B76] Liu X. Liu Q. Kong F. Qiao X. Xu Z. (2018). Molecularly imprinted fluorescent probe based on hydrophobic CdSe/ZnS quantum dots for the detection of methamidophos in fruit and vegetables. Adv. Polym. Technol. 37, 1790–1796. 10.1002/adv.21838

[B77] Liu Z. Lu Y. Shi Y. Wang P. Jones K. Sweetman A. J.. (2017). Crop bioaccumulation and human exposure of perfluoroalkyl acids through multi-media transport from a mega fluorochemical industrial park, China. Environ. Int. 106, 37–47. 10.1016/j.envint.2017.05.014 28558301

[B78] Lobato A. Pereira E. A. Gonçalves L. M. (2020). Combining capillary electromigration with molecular imprinting techniques towards an optimal separation and determination. Talanta 221:121546 10.1016/j.talanta.2020.121546 33076105

[B79] Lu Y. Song S. Wang R. Liu Z. Meng J. Sweetman A. J.. (2015). Impacts of soil and water pollution on food safety and health risks in China. Environ. Int. 77, 5–15. 10.1016/j.envint.2014.12.010 25603422

[B80] Lucci P. Moret S. Bettin S. Conte L. (2017). Selective solid-phase extraction using a molecularly imprinted polymer for the analysis of patulin in apple-based foods. J. Sep. Sci. 40, 458–465. 10.1002/jssc.201601009 27805312

[B81] Lulinski P. (2017). Molecularly imprinted polymers based drug delivery devices: a way to application in modern pharmacotherapy. a review. Mater. Sci. Eng. C Mater. Biol. Appl. 76, 1344–1353. 10.1016/j.msec.2017.02.138 28482502

[B82] Ma Y. Xu S. Wang S. Wang L. (2015). Luminescent molecularly-imprinted polymer nanocomposites for sensitive detection. Trends Anal. Chem. 67, 209–216. 10.1016/j.trac.2015.01.012

[B83] Mahmoudpour M. Torbati M. Mousavi M.-M. de la Guardia M. Ezzati Nazhad Dolatabadi J. (2020). Nanomaterial-based molecularly imprinted polymers for pesticides detection: recent trends and future prospects. Trends Anal. Chem. 129:115943 10.1016/j.trac.2020.115943

[B84] Mirzajani R. Kardani F. Ramezani Z. (2020). Fabrication of UMCM-1 based monolithic and hollow fiber - Metal-organic framework deep eutectic solvents/molecularly imprinted polymers and their use in solid phase microextraction of phthalate esters in yogurt, water and edible oil by GC-FID. Food Chem. 314:126179. 10.1016/j.foodchem.2020.126179 31968292

[B85] Moreno-Gonzalez D. Jac P. Riasova P. Novakova L. (2020). In-line molecularly imprinted polymer solid phase extraction-capillary electrophoresis coupled with tandem mass spectrometry for the determination of patulin in apple-based food. Food Chem. 334:127607. 10.1016/j.foodchem.2020.127607 32711279

[B86] Nezhadali A. Senobari S. Mojarrab M. (2016). 1,4-dihydroxyanthraquinone electrochemical sensor based on molecularly imprinted polymer using multi-walled carbon nanotubes and multivariate optimization method. Talanta 146, 525–532. 10.1016/j.talanta.2015.09.016 26695300

[B87] Ning F. Qiu T. Wang Q. Peng H. Li Y. Wu X.. (2017). Dummy-surface molecularly imprinted polymers on magnetic graphene oxide for rapid and selective quantification of acrylamide in heat-processed (including fried) foods. Food Chem. 221, 1797–1804. 10.1016/j.foodchem.2016.10.101 27979164

[B88] Pandey H. Khare P. Singh S. Singh S. P. (2020). Carbon nanomaterials integrated molecularly imprinted polymers for biological sample analysis: a critical review. Mater. Chem. Phys. 239:121966 10.1016/j.matchemphys.2019.121966

[B89] Pataer P. Muhammad T. Turahun Y. Yang W. Aihebaier S. Wubulikasimu M.. (2019). Preparation of a stoichiometric molecularly imprinted polymer for auramine O and application in solid-phase extraction. J. Sep. Sci. 42, 1634–1643. 10.1002/jssc.201801234 30756481

[B90] Piletska E. V. Abd B. H. Krakowiak A. S. Parmar A. Pink D. L. Wall K. S.. (2015). Magnetic high throughput screening system for the development of nano-sized molecularly imprinted polymers for controlled delivery of curcumin. Analyst 140, 3113–3120. 10.1039/C4AN02292K 25751126

[B91] Piletsky S. Canfarotta F. Poma A. Bossi A. M. Piletsky S. (2020). Molecularly imprinted polymers for cell recognition. Trends Biotechnol. 38, 368–387. 10.1016/j.tibtech.2019.10.002 31677857

[B92] Płotka-Wasylka J. Szczepańska N. de la Guardia M. Namieśnik J. (2016). Modern trends in solid phase extraction: New sorbent media. Trends Anal. Chem. 77, 23–43. 10.1016/j.trac.2015.10.010

[B93] Qian K. Deng Q. Fang G. Wang J. Pan M. Wang S.. (2016). Metal-organic frameworks supported surface-imprinted nanoparticles for the sensitive detection of metolcarb. Biosens. Bioelectron. 79, 359–363. 10.1016/j.bios.2015.12.071 26735869

[B94] Qu Y. Qin L. Liu X. Yang Y. (2020). Reasonable design and sifting of microporous carbon nanosphere-based surface molecularly imprinted polymer for selective removal of phenol from wastewater. Chemosphere 251:126376. 10.1016/j.chemosphere.2020.126376 32169694

[B95] Ren X. Zeng G. Tang L. Wang J. Wan J. Liu Y.. (2018). Sorption, transport and biodegradation - An insight into bioavailability of persistent organic pollutants in soil. Sci. Total Environ. 610–611, 1154–1163. 10.1016/j.scitotenv.2017.08.089 28847136

[B96] Rodriguez K. J. Pellizzoni M. M. Chadwick R. J. Guo C. Bruns N. (2019). Enzyme-initiated free radical polymerizations of vinyl monomers using horseradish peroxidase. Methods Enzymol. 627, 249–262. 10.1016/bs.mie.2019.08.013 31630743

[B97] Rudd N. D. Wang H. Fuentes-Fernandez E. M. Teat S. J. Chen F. Hall G.. (2016). Highly efficient luminescent metal-organic framework for the simultaneous detection and removal of heavy metals from water. ACS Appl. Mater. Interfaces 8, 30294–30303. 10.1021/acsami.6b10890 27736058

[B98] Rutkowska M. Płotka-Wasylka J. Morrison C. Wieczorek P. P. Namieśnik J. Marć M. (2018). Application of molecularly imprinted polymers in analytical chiral separations and analysis. Trends Anal. Chem. 102, 91–102. 10.1016/j.trac.2018.01.011

[B99] Sharma P. S. D'Souza F. Kutner W. (2012). Molecular imprinting for selective chemical sensing of hazardous compounds and drugs of abuse. Trends Anal. Chem. 34, 59–77. 10.1016/j.trac.2011.11.005

[B100] Singh M. Singh S. Singh S. P. Patel S. S. (2020). Recent advancement of carbon nanomaterials engrained molecular imprinted polymer for environmental matrix. Trends Environ. Anal. Chem. 27:e00092 10.1016/j.teac.2020.e00092

[B101] Sonawane S. L. Asha S. K. (2017). Probing cavity versus surface preference of fluorescent template molecules in molecularly imprinted polystyrene microspheres. J. Polym. Sci. Part A Polym. Chem. 55, 1558–1565. 10.1002/pola.28523

[B102] Song Y. P. Zhang L. Wang G. N. Liu J. X. Liu J. Wang J. P. (2017). Dual-dummy-template molecularly imprinted polymer combining ultra performance liquid chromatography for determination of fluoroquinolones and sulfonamides in pork and chicken muscle. Food Control 82, 233–242. 10.1016/j.foodcont.2017.07.002

[B103] Song X. Xu S. Chen L. Wei Y. Xiong H. (2014). Recent advances in molecularly imprinted polymers in food analysis. J. Appl. Polym. Sci. 131:40766 10.1002/app.40766

[B104] Söylemez M. A. Güven O. Barsbay M. (2018). Method for preparing a well-defined molecularly imprinted polymeric system via radiation-induced RAFT polymerization. Eur. Polym. J. 103, 21–30. 10.1016/j.eurpolymj.2018.03.037

[B105] Sposito A. J. Kurdekar A. Zhao J. Hewlett I. (2018). Application of nanotechnology in biosensors for enhancing pathogen detection. Wiley Interdiscip. Rev. Nanomed. Nanobiotechnol. 10:e1512. 10.1002/wnan.1512 29528198

[B106] Sun X. Peng J. Wang M. Wang J. Tang C. Yang L.. (2018). Determination of nine bisphenols in sewage and sludge using dummy molecularly imprinted solid-phase extraction coupled with liquid chromatography tandem mass spectrometry. J. Chromatogr. A 1552, 10–16. 10.1016/j.chroma.2018.04.004 29678407

[B107] Svitkova V. Palchetti I. (2020). Functional polymers in photoelectrochemical biosensing. Bioelectrochemistry 136:107590. 10.1016/j.bioelechem.2020.107590 32674004

[B108] Tang K. Gu X. Luo Q. Chen S. Wu L. Xiong J. (2014). Preparation of molecularly imprinted polymer for use as SPE adsorbent for the simultaneous determination of five sulphonylurea herbicides by HPLC. Food Chem. 150, 106–112. 10.1016/j.foodchem.2013.10.152 24360426

[B109] Tang Y. Li M. Gao X. Liu X. Ma Y. Li Y. (2015). Preconcentration of the antibiotic enrofloxacin using a hollow molecularly imprinted polymer, and its quantitation by HPLC. Microchim. Acta 183, 589–596. 10.1007/s00604-015-1681-7

[B110] Tarannum N. Khatoon S. Dzantiev B. B. (2020). Perspective and application of molecular imprinting approach for antibiotic detection in food and environmental samples: a critical review. Food Control 118:107381 10.1016/j.foodcont.2020.107381

[B111] Turiel E. Martin-Esteban A. (2010). Molecularly imprinted polymers for sample preparation: a review. Anal. Chim. Acta 668, 87–99. 10.1016/j.aca.2010.04.019 20493285

[B112] Wang H. Liu Y. Yao S. Zhu P. (2018). Selective recognization of dicyandiamide in bovine milk by mesoporous silica SBA-15 supported dicyandiamide imprinted polymer based on surface molecularly imprinting technique. Food Chem. 240, 1262–1267. 10.1016/j.foodchem.2017.08.066 28946251

[B113] Wang J. Gao L. Han D. Pan J. Qiu H. Li H.. (2015). Optical detection of lambda-cyhalothrin by core-shell fluorescent molecularly imprinted polymers in Chinese spirits. J. Agric. Food Chem 63, 2392–2399. 10.1021/jf5043823 25632984

[B114] Wang S. Meng X. Zhou H. Liu Y. Secundo F. Liu Y. (2016). Enzyme stability and activity in non-aqueous reaction systems: a mini review. Catalysts 6:32 10.3390/catal6020032

[B115] Wang W. Xu Y. Liu X. Peng L. Huang T. Yan Y. (2020). Efficient fabrication of ratiometric fluorescence imprinting sensors based on organic-inorganic composite materials and highly sensitive detection of oxytetracycline in milk. Microchem. J. 157:105063 10.1016/j.microc.2020.105053

[B116] Wang Y. Zhou J. Zhang B. Tian L. Ali Z. Zhang Q. (2017). Fabrication and characterization of glutathione-imprinted polymers on fibrous SiO 2 microspheres with high specific surface. Chem. Eng. J. 327, 932–940. 10.1016/j.cej.2017.06.184

[B117] Wang Y. L. Gao Y. L. Wang P. P. Shang H. Pan S. Y. Li X. J. (2013). Sol-gel molecularly imprinted polymer for selective solid phase microextraction of organophosphorous pesticides. Talanta 115, 920–927. 10.1016/j.talanta.2013.06.056 24054683

[B118] Wang Z. Yan R. Liao S. Miao Y. Zhang B. Wang F. (2018). *In situ* reduced silver nanoparticles embedded molecularly imprinted reusable sensor for selective and sensitive SERS detection of Bisphenol A. Appl. Surf. Sci. 457, 323–331. 10.1016/j.apsusc.2018.06.283

[B119] Wei S. Hu X. Liu H. Wang Q. He C. (2015). Rapid degradation of Congo red by molecularly imprinted polypyrrole-coated magnetic TiO_2_ nanoparticles in dark at ambient conditions. J. Hazard. Mater. 294, 168–176. 10.1016/j.jhazmat.2015.03.067 25867589

[B120] Wu X. Zhang Z. Li J. You H. Li Y. Chen L. (2015). Molecularly imprinted polymers-coated gold nanoclusters for fluorescent detection of bisphenol A. Sens. Actuat. B Chem. 211, 507–514. 10.1016/j.snb.2015.01.115

[B121] Wulff G. (2002). Enzyme-like catalysis by molecularly imprinted polymers. Am. Chem. Soc. 102, 1–25. 10.1021/cr980039a 11782127

[B122] Wulff G. Vesper W. Grobe-Einsler R. Sarhan A. (1977). On the synthesis of polymers containing chiral cavities and their use for the resolution of racemates. Makromol. Chem. 178, 2799–2816. 10.1002/macp.1977.021781004

[B123] Xiao J. Xu X. Wang F. Ma J. Liao M. Shi Y.. (2019). Analysis of exposure to pesticide residues from traditional Chinese medicine. J. Hazard. Mater. 365, 857–867. 10.1016/j.jhazmat.2018.11.075 30497040

[B124] Xie T. Zhang M. Chen P. Zhao H. Yang X. Yao L. (2017). A facile molecularly imprinted electrochemical sensor based on graphene: application to the selective determination of thiamethoxam in grain. RSC Adv. 7, 38884–38894. 10.1039/C7RA05167K

[B125] Xu L. Fang G. Pan M. Wang X. Wang S. (2016). One-pot synthesis of carbon dots-embedded molecularly imprinted polymer for specific recognition of sterigmatocystin in grains. Biosens. Bioelectron. 77, 950–956. 10.1016/j.bios.2015.10.072 26544869

[B126] Xu S. Chen L. Li J. Qin W. Ma J. (2011). Preparation of hollow porous molecularly imprinted polymers and their applications to solid-phase extraction of triazines in soil samples. J. Mater. Chem. 21, 12047–12053. 10.1039/c1jm10905g

[B127] Yu H. He Y. She Y. Wang M. Yan Z. Ren J. H.. (2019). Preparation of molecularly imprinted polymers coupled with high-performance liquid chromatography for the selective extraction of salidroside from Rhodiola crenulata. J. Chromatogr. B Analyt. Technol. Biomed. Life Sci. 1118–1119, 180–186. 10.1016/j.jchromb.2019.04.004 31054452

[B128] Yu J. Wang X. Kang Q. Li J. Shen D. Chen L. (2017). One-pot synthesis of a quantum dot-based molecular imprinting nanosensor for highly selective and sensitive fluorescence detection of 4-nitrophenol in environmental waters. Environ. Sci. Nano 4, 493–502. 10.1039/C6EN00395H

[B129] Yuan Y. Yang Y. Faheem M. Zou X. Ma X. Wang Z.. (2018a). molecularly imprinted porous aromatic frameworks serving as porous artificial enzymes. Adv. Mater. Weinheim 30:e1800069. 10.1002/adma.201800069 29782674

[B130] Yuan Y. Yang Y. Ma X. Meng Q. Wang L. Zhao S.. (2018b). molecularly imprinted porous aromatic frameworks and their composite components for selective extraction of uranium ions. Adv. Mater. 30:e1706507. 10.1002/adma.201706507 29423920

[B131] Zeng H. Wang Y. Nie C. Kong J. Liu X. (2012). Preparation of magnetic molecularly imprinted polymers for separating rutin from Chinese medicinal plants. Analyst 137, 2503–2512. 10.1039/c2an35259a 22489285

[B132] Zhang L. Zhu C. Chen C. Zhu S. Zhou J. Wang M.. (2018). Determination of kanamycin using a molecularly imprinted SPR sensor. Food Chem. 266, 170–174. 10.1016/j.foodchem.2018.05.128 30381173

[B133] Zhang M. Zhao H. T. Yang X. Zhang W. T. Wang J. Liu G. Y. (2016). Preparation and characterization of surface molecularly imprinted film coated on a magnetic nanocore for the fast and selective recognition of the new neonicotinoid insecticide paichongding (IPP). RSC Adv. 6, 3714–3722. 10.1039/C5RA22138B

[B134] Zhang R.-R. Zhan J. Xu J.-J. Chai J.-Y. Zhang Z.-M. Sun A.-L. (2020). Application of a novel electrochemiluminescence sensor based on magnetic glassy carbon electrode modified with molecularly imprinted polymers for sensitive monitoring of bisphenol A in seawater and fish samples. Sens. Actuat B Chem. 317:128237 10.1016/j.snb.2020.128237

[B135] Zhang Z. Li Y. Zhang X. Liu J. (2019). Molecularly imprinted nanozymes with faster catalytic activity and better specificity. Nanoscale 11, 4854–4863. 10.1039/C8NR09816F 30820498

[B136] Zhao W.-R. Kang T.-F. Xu Y.-H. Zhang X. Liu H. Ming A.-J. (2020). Electrochemiluminescence solid-state imprinted sensor based on graphene/CdTe@ZnS quantum dots as luminescent probes for low-cost ultrasensing of diethylstilbestrol. Sens. Actuat. B Chem. 306:127563 10.1016/j.snb.2019.127563

[B137] Zhao Z. Fan J. Wang C. Cheng B. Xue Y. Yin S. (2017). Preparation and properties of phenol imprinted polymers based on silica modified multi-walled carbon nanotubes. J. Nanosci. Nanotechnol. 17, 1504–1509. 10.1166/jnn.2017.12651 29688662

[B138] Zhou J. W. Zou X. M. Song S. H. Chen G. H. (2018). Quantum dots applied to methodology on detection of pesticide and veterinary drug residues. J. Agric. Food Chem. 66, 1307–1319. 10.1021/acs.jafc.7b05119 29378133

[B139] Zhu R. Lai M. Zhu M. Liang H. Zhou Q. Li R.. (2020). A functional ratio fluorescence sensor platform based on the graphene/Mn-ZnS quantum dots loaded with molecularly imprinted polymer for selective and visual detection sinapic acid. Spectrochim. Acta A Mol. Biomol. Spectrosc. 244:118845. 10.1016/j.saa.2020.118845 32882656

